# New Sesquiterpenenoids from *Ainsliaea yunnanensis*

**DOI:** 10.3390/molecules21081031

**Published:** 2016-08-08

**Authors:** Xiang-Lei Wu, Xiao-Juan Xiong, Wen-Quan Lu, Hao Huang, Yun-Heng Shen, Zhi-Jun Wu, Wan-Sheng Chen

**Affiliations:** 1 Department of Pharmacy, Changzheng Hospital, Second Military Medical University, 415 Fengyang Road, Shanghai 200003, China; 15921754936@163.com (X.-L.W.); lwqp@163.com (W.-Q.L.); 2 College of Chemical and Biological Engineering, Yichun University, 576 Xuefu Road, Yichun 336000, China; ycxxxj@163.com (X.-J.X.); jxychh2008@163.com (H.H.); 3 College of Pharmacy, Second Military Medical University, 325 Guohe Road, Shanghai 200433, China; shenyunheng@hotmail.com

**Keywords:** *Ainsliaea yunnanensis*, *Ainsliaea*, sesquiterpenoid, anti-inflammatory activity

## Abstract

Investigation of the ethanol extract of the whole plant of *Ainsliaea yunnanensis* led to the isolation of four new dimeric sesquiterpene lactones, ainsliadimer F–I (**1**–**4**), together with seven known dimeric sesquiterpene lactones (**5**–**11**) and ten sesquiterpenes (**12**–**21**). Their structures were elucidated by spectroscopic methods. The relative stereochemistry of ainsliadimer F was further confirmed by single crystal X-ray diffraction analysis. Compounds **1**–**21** were tested for the inhibition of nuclear factor kappa B (NF-*κ*B) in the 293-NF-*κ*B-luciferase reporter cell line induced by lipopolysaccharide (LPS), and Compounds **5**, **18**, **20** and **21** were further tested for the production of TNF-*α*, IL-1*β*, IL-6 and IL-10 in RAW 264.7 macrophages induced by LPS. Compounds **5**, **18**, **20** and **21** exhibited significant activity in anti-inflammatory activity assays.

## 1. Introduction

The plants of Asteraceae are well known to contain biologically-active sesquiterpenoids [1,2,3,4]. Within this family, the genus Ainsliaea comprises 70 species, 48 of which are indigenous to China. Phytochemical studies have indicated that sesquiterpenoids are characteristic constituents of Ainsliaea species [5,6,7,8], and guaiane-type sesquiterpene, as one of the most important chemical types of sesquiterpenes, has attracted much interest due to the structural diversity and varied biological activities, such as anti-inflammatory [9] and antitumor [10,11]. *A. yunnanensis*, a plant of Ainsliaea, is mainly distributed in Yunnan province of China, which has been used in Chinese folk medicine to treat rheumatism, lumbago and gonitis [12]. Some chemical constituents of this plant have been reported previously [12,13].

**Figure 1:**
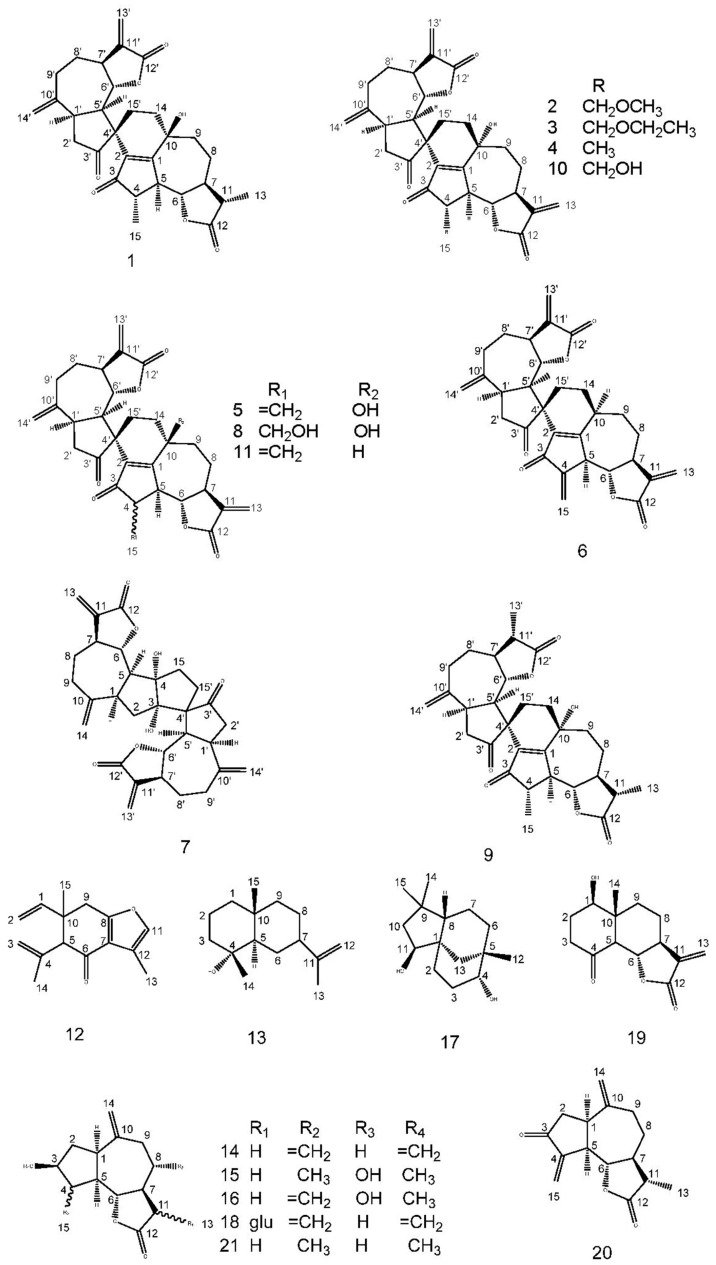
Structures of Compounds **1**–**21**.

As part of the consecutive study of Ainsliaea species, our investigation on *A. yunnanensis* led to the isolation of four new dimeric sesquiterpene lactones, ainsliadimer F (**1**), ainsliadimer G (**2**), ainsliadimer H (**3**) and ainsliadimer I (**4**), together with seven known dimeric sesquiterpene lactones, gochnatiolide A (**5**) [14], gochnatiolide B (**6**) [15], ainsliadimer A (**7**) [16], ainsliadimer B (**8**) [15], ainsliadimer C (**9**) [17], ainsliadimer D (**10**) [17] and ainsliadimer E (**11**) [18], and ten sesquiterpenes, curzerenone (**12**) [19], selin-11-en-4 alpha-ol (**13**) [20], zaluzanin C (**14**) [21], isolipidiol (**15**) [22], 11,13-dihydro-deacylcynaropicrin (**16**) [23], clovane-2 beta, 9 alpha-diol (**17**) [24], glucozaluzanin C (**18**) [25], ainsliatone A (**19**) [26], dihydroestafiatone (**20**) [27] and dihydroestafiatol (**21**) [27]. Their structures were elucidated by spectroscopic methods and by comparison with the literature. Their structures were show in Figure 1. These compounds were tested for anti-inflammatory activities, and Compounds **5**, **18**, **20** and **21** exhibited significant activities in anti-inflammatory activity assays.

## 2. Results and Discussion

### 2.1. Structure Elucidation of New Compounds

**Figure 2:**
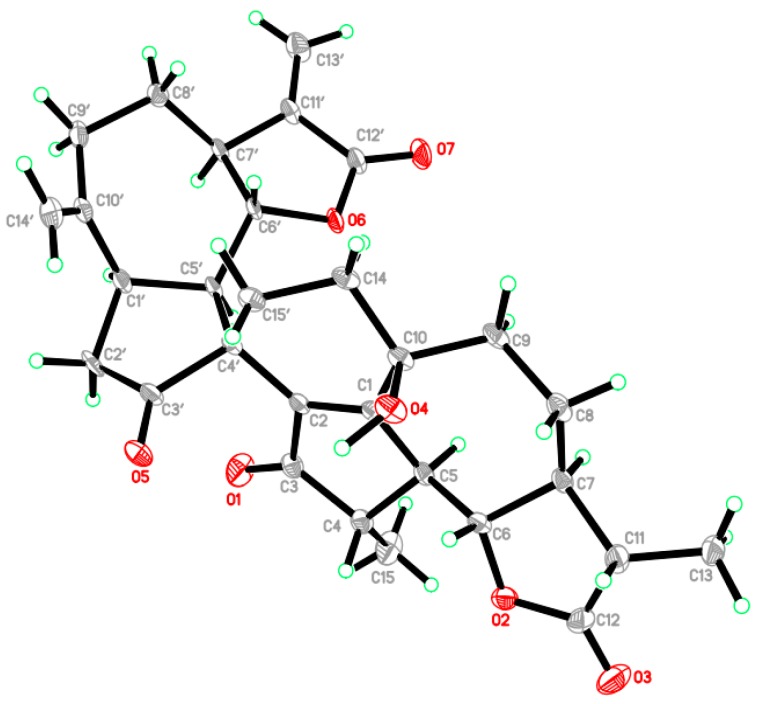
Single-crystal X-ray structure of Compound **1**.

Compound **1** was obtained as colorless prisms, and its molecular formula was established to be C_30_H_34_O_7_ by its HRESI (*m*/*z* 529.2206 [M + Na]^+^) and ^13^C-NMR spectra. The ^13^C-NMR spectrum exhibited 30 carbon resonances, classified into ten quaternary carbons, nine methines, including two oxygen-substituted carbons, nine methylenes and two methyls. The signals at *δ*_C_ 208.5, 222.3 were ascribed to two ketone groups. The characteristic signals at *δ*_C_ 12.6, 14.3 were ascribed to two methyls; the signals at *δ*_C_ 114.1, 121.6, 138.5 and 150.1 were ascribed to two exocyclic double bonds; the signals at *δ*_C_ 169.3, 178.6 were ascribed to two ester carbonyls; and the signals at *δ*_C_ 82.6, 83.9 were ascribed to two oxygen-bearing methines. In the ^1^H-NMR spectrum, the signals at *δ*_H_ 1.27 (3H, d, *J* = 6.9 Hz) and *δ*_H_ 1.27 (3H, d, *J* = 7.1 Hz) implied the presence of two methyls; the signals at *δ*_H_ 4.72 (1H, brs), 5.09 (1H, brs), 5.58 (1H, d, *J* = 3.0 Hz) and 6.26 (1H, d, *J* = 3.4 Hz) implied the presence of two exocyclic double bonds; the signals at *δ*_H_ 4.16 (1H, t, *J* = 9.4 Hz) and 4.32 (1H, dd, *J* = 11.0, 9.6 Hz) implied the presence of two oxygen-bearing methines. The ^1^H- and ^13^C-NMR spectra of Compound **1** indicated the presence of two guaianolide structure moieties. Comparison of the NMR data (Table 1) with those of the known compound gochnatiolide A (**5**) showed close resemblances [14], except that two exocyclic double bonds at C-4/C-15 and C-11/C-13 in gochnatiolide A were replaced by two methyls and two methines in Compound **1**. This was confirmed by HMBC correlations of Me-15 (*δ*_H_ 1.27) with C-3 (*δ*_C_ 208.5), C-4 (*δ*_C_ 47.0), C-5 (*δ*_C_ 54.8) and Me-13 (*δ*_H_ 1.27) with C-7 (*δ*_C_ 54.9), C-11 (*δ*_C_ 41.9) and C-12 (*δ*_C_ 178.6). The relative configuration of Me-15 and Me-13 were deduced to be *α*-oriented based on the NOESY correlations of Me-15 with H-5*α* (*δ*_H_ 2.80) and Me-13 with H-7*α* (*δ*_H_ 1.84). The structure and relative configuration of **1** were further confirmed by the X-ray diffraction analysis (Figure 2). Compound **1** was named as ainsliadimer F.

Compound **2** was isolated as colorless prisms. The molecular formula of Compound **2** was determined as C_31_H_34_O_8_ from the quasi-molecular ion peak [M + Na]^+^ at *m*/*z* 557.2148 in its positive HRESI. The ^13^C-NMR spectrum exhibited 31 carbon resonances, classified into eleven quaternary carbons, eight methines, including two oxygen-substituted carbons, eleven methylenes and one methoxyl group. The signals at **δ**_C_ 206.3, 222.5 were ascribed to two ketone groups. The signals at *δ*_C_ 170.1, 169.4 were ascribed to two ester carbonyls. The signals at *δ*_C_ 114.1, 119.2, 121.5, 138.6, 139.5 and 150.2 were ascribed to three exocyclic double bonds; the signals at *δ*_C_ 83.9, 82.6 were ascribed to two oxygen-substituted methines; the signal at *δ*_C_ 68.4 was linked to one oxygen atom; and the signal at *δ*_C_ 59.2 was ascribed to one methoxyl group. Analysis of NMR data (Table 1) revealed a dimeric sesquiterpene skeleton consisting of guaianolide moieties, and it was quite close to the known compound ainsliadimer D (**10**) [17], except for a methoxy group in Compound **2**, replacing the hydroxyl group in ainsliadimer D. This was confirmed by the HMBC correlations of C-1°′(*δ*_C_ 59.2) with H-15 (*δ*_H_ 3.94, 3.74). The structure of **2** was established on the basis of 1D- and 2D-NMR spectra (^1^H, ^13^C, DEPT, COSY, HSQC and HMBC). Accordingly, the structure of Compound **2** was named as ainsliadimer G. Compound **2** was a natural chemical constituent and not an isolation artifact, which was tested by HPLC (Figure S30) in a crude extract.

Compound **3** was obtained as a colorless powder and showed the [M + Na]^+^ ion peak at *m*/*z* 571.2310 in its HRESI, corresponding to a molecular formula of C_32_H_36_O_8_. The ^13^C-NMR spectrum showed 32 carbon resonances, classified into eleven quaternary carbons, eight methines, including two oxygen-substituted carbons, twelve methylenes, including two oxygen-bearing carbons, and one methyl. Analysis of NMR data (Table 1) showed it to be quite close to Compound **2**, except for an ethoxy group in Compound **3** replacing a methoxy group in Compound **2**. This was confirmed by HMBC correlations of H-15 (*δ*_H_ 3.96, 3.76) with C-1″ (*δ*_C_ 66.8). Accordingly, the structure of Compound **3** was named as ainsliadimer H. Compound **3** was a natural chemical constituent, which was also tested by HPLC (Figure S31) in a crude extract.

Compound **4** was obtained as colorless prisms, and its molecular formula was established to be C_30_H_32_O_7_ by its HRESI (*m*/*z* 527.2043 [M + Na]^+^) and ^13^C-NMR spectra. The ^13^C-NMR spectrum exhibited 30 carbon resonances, classified into eleven quaternary carbons, eight methines, including two oxygen-substituted carbons, ten methylenes and one methyl. The ^1^H- and ^13^C-NMR spectra of **4** were quite close to those of ainsliadimer D (**10**) [17], except for one hydroxymethyl at the position C-4 in ainsliadimer D was replaced by one methyl in Compound **4**. This was confirmed by HMBC correlations of Me-15 (*δ*_H_ 1.30) with C-3 (*δ*_C_ 208.4), C-4 (*δ*_C_ 47.0), C-5 (*δ*_C_ 55.1). The structure of **4** was established on the basis of 1D- and 2D-NMR spectra and was named ainsliadimer I.

In addition to four new dimeric sesquiterpene lactones (**1**–**4**), seventeen known sesquiterpenes, identified as gochnatiolide A (**5**), gochnatiolide B (**6**), ainsliadimer A (**7**), ainsliadimer B (**8**), ainsliadimer C (**9**), ainsliadimer D (**10**), ainsliadimer E (**11**), curzerenone (**12**), selin-11-en-4 alpha-ol (**13**), zaluzanin C (**14**), isolipidiol (**15**), 11,13-dihydro-deacylcynaropicrin (**16**), clovane-2 beta, 9 alpha-diol (**17**), glucozaluzanin C (**18**), ainsliatone A (**19**), dihydroestafiatone (**20**) and dihydroestafiatol (**21**). These compounds were identified by spectral analysis, and we found that their spectral data were consistent with the spectroscopic data reported in the corresponding literature.

### 2.2. Chemotaxonomic Significance

The present phytochemical investigation on this plant led to the isolation of twenty-one sesquiterpenoids from *A. yunnanensis*, including eighteen guaiane-type sesquiterpene lactones (**1**–**11**, **14**–**16** and **18**–**21**), one bicyclic sesquiterpane (**12**), one eudesmane sesquiterpene (**13**) and one clovane sesquiterpene (**17**). The general chemotaxonomic pattern of *A. yunnanensis* is similar to other Ainsliaea species in terms of chemical classes; this is the first report of Compounds **5**, **6**, **7**–**10**, **14**–**16**, **18**–**19** from *A. yunnanensis*, and the first report of Compounds **1**–**4**, **11**–**13**, **17**, **21**–**21** from the genus Ainsliaea.

The guaiane-type sesquiterpenes have wide-spread occurrence in the genus Ainsliaea species. Among them, Compounds **1**–**11** were dimeric guaiane-type sesquiterpene lactones; they were used as important chemotaxonomic markers in this genus [15,16,17]. They are generally biosynthesized from two monomeric sesquiterpene lactones through a six-membered cyclohexene of the 3,4-dihydro-2*H*-pyran ring via hetero Diels-Alder cycloaddition reactions, except ainsliadimer A (**7**), which represents the single dimeric sesquiterpene lactone with a five-membered cyclopentane ring [16]. On the other hand, Compound **11** has been reported to be present in the genera Gochnatia; we now report its presence for the first time in the Ainsliaea family, although the other dimeric guaiane-type sesquiterpene lactones were only isolated from Ainsliaea species. This is an interesting finding, as Gochnatia could be an alternative source of these compounds. 

To conclude, the isolation of different sesquiterpene lactones is consistent with the chemical profile of the other Ainsliaea species. Of these, dimeric guaiane-type sesquiterpene lactones structurally characterized by a six-membered ring are systematically important and might serve as the common characteristic constituents of both Ainsliaea and Asteraceae families.

### 2.3. Evaluation of Biological Activity

**Figure 3:**
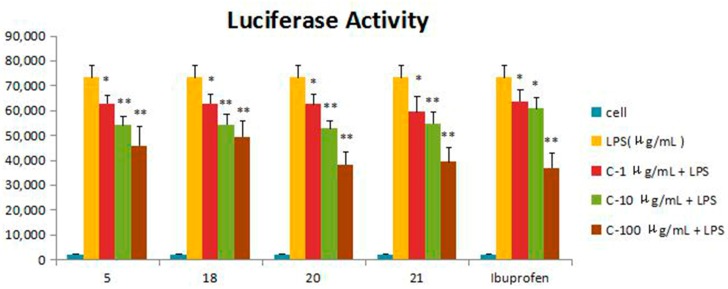
Inhibitory effects of Compounds **5**, **18**, **20** and **21** (1, 10, 100 μg/mL) on NF-*κ*B in the luciferase activity assay. Data are expressed as the mean ± S.E.M. of three independent experiments. Cell: cultures were not exposed to lipopolysaccharide (LPS); LPS: cultures were subjected to LPS; LPS + Drug: compounds were added to the cultures during LPS; Positive control: LPS + Ibuprofen. ∗ *p* < 0.05 vs. LPS; ∗∗ *p* < 0.01 vs. LPS.

**Figure 4:**
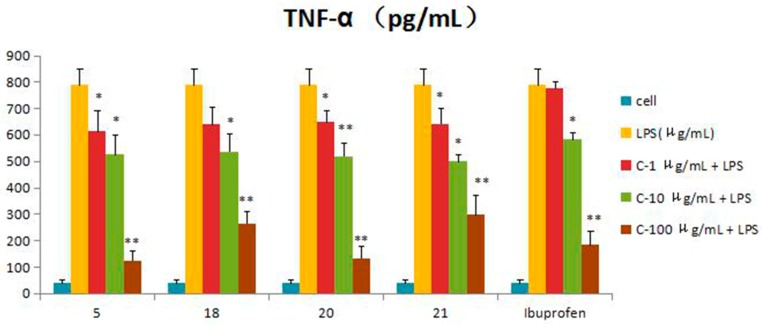
Inhibitory effects of Compounds **5**, **18**, **20** and **21** (1, 10, 100 μg/mL) on TNF-*α* production stimulated by LPS (10 μg/mL) in RAW 264.7 cells (mouse leukemic monocyte macrophage cell line). Data are expressed as the mean ± S.E.M. of three independent experiments. Cell: cultures were not exposed to lipopolysaccharide (LPS); LPS: cultures were subjected to LPS; LPS + Drug: compounds were added to the cultures during LPS; Positive control: LPS + Ibuprofen. ∗ *p* < 0.05 vs. LPS; ∗∗ *p* < 0.01 vs. LPS.

**Figure 5:**
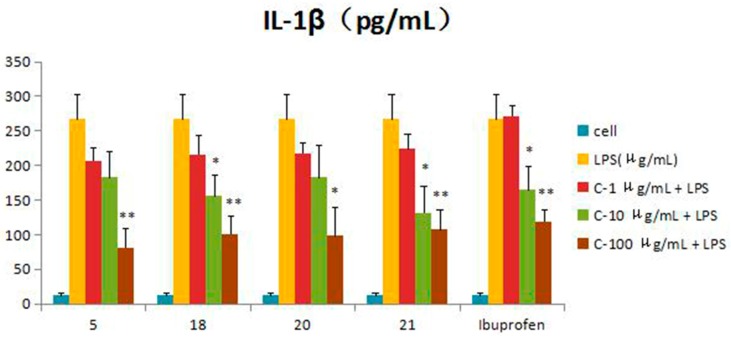
Inhibitory effects of Compounds **5**, **18**, **20** and **21** (1, 10, 100 μg/mL) on IL-1*β* production stimulated by LPS (10 μg/mL) in RAW 264.7 cells (mouse leukemic monocyte macrophage cell line). Data are expressed as the mean ± S.E.M. of three independent experiments. Cell: cultures were not exposed to lipopolysaccharide (LPS); LPS: cultures were subjected to LPS; LPS + Drug: compounds were added to the cultures during LPS; Positive control: LPS + Ibuprofen. ∗ *p* < 0.05 vs. LPS; ∗∗ *p* < 0.01 vs. LPS.

**Figure 6:**
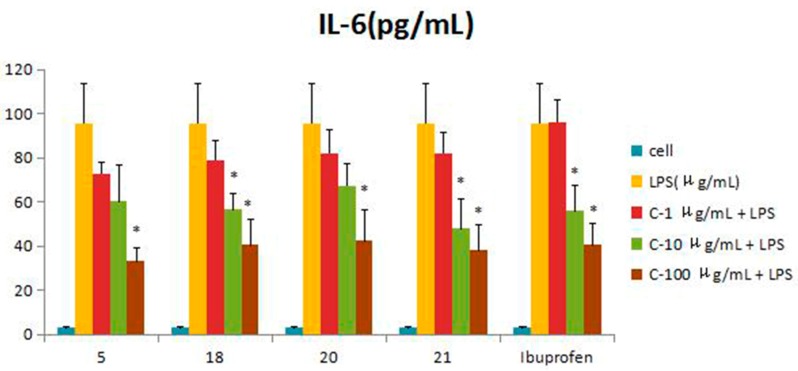
Inhibitory effects of Compounds **5**, **18**, **20** and **21** (1, 10, 100 μg/mL) on IL-6 production stimulated by LPS (10 μg/mL) in RAW 264.7 cells (mouse leukemic monocyte macrophage cell line). Data are expressed as the mean ± S.E.M. of three independent experiments. Cell: cultures were not exposed to lipopolysaccharide (LPS); LPS: cultures were subjected to LPS; LPS + Drug: compounds were added to the cultures during LPS; Positive control: LPS + Ibuprofen. ∗ *p* < 0.05 vs. LPS.

**Figure 7:**
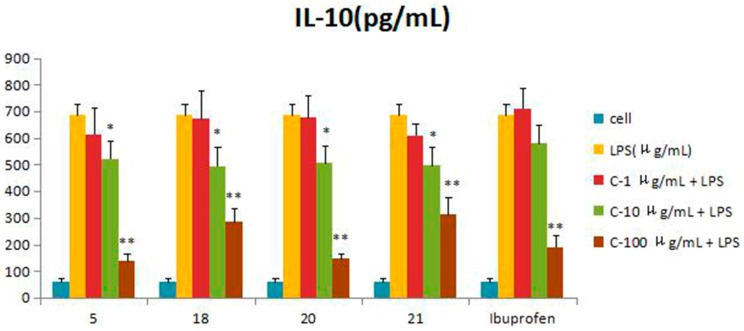
Inhibitory effects of Compounds **5**, **18**, **20** and **21** (1, 10, 100 μg/mL) on IL-10 production stimulated by LPS (10 μg/mL) in RAW 264.7 cells (mouse leukemic monocyte macrophage cell line). Data are expressed as the mean ± S.E.M. of three independent experiments. Cell: cultures were not exposed to lipopolysaccharide (LPS); LPS: cultures were subjected to LPS; LPS + Drug: compounds were added to the cultures during LPS; Positive control: LPS + Ibuprofen. ∗ *p* < 0.05 vs. LPS; ∗∗ *p* < 0.01 vs. LPS.

Compounds **1**–**21** were assessed for inhibitory activity in a luciferase assay. Compared to the cell group, the luciferase activity of the LPS group was significantly enhanced, which indicated that the inflammatory cell model induced by LPS was constructed successfully. Then, we found that the luciferase activity of Compounds **5**, **18**, **20** and **21** was significantly decreased by comparing to the LPS group, which showed that they had significant inhibitory effect on NF-*κ*B activity. The effects of Compounds **5**, **18**, **20** and **21** on the inflammatory response were investigated further. The anti-inflammatory effects were evaluated by investigating the inhibitory activity of the compounds on the production of TNF-*α*, IL-1*β*, IL-6 and IL-10 in RAW 264.7 macrophages induced by LPS. For all assays, ibuprofen was used as a positive control. Compared to the LPS group, these compounds were tested their inhibitory ratio of TNF-*α*, IL-1β, IL-6 and IL-10 release in LPS-stimulated RAW 264.7 macrophages in vitro at three different concentration (1 μg/mL, 10 μg/mL, 100 μg/mL). The concentration of TNF-*α* in the RAW 264.7 cells pretreated with Compounds **5**, **18**, **20** and **21** was reduced to 67.9%, 66.5%, 65.7% and 63.2% at 10 μg/mL and 33%, 15.5%, 16.9% and 37.7% at 100 μg/mL, respectively, while the inhibitory rates of the positive control ibuprofen were 73.8% and 23.0%. In the same way, the concentrations of IL-1*β*, IL-6 and IL-10 in the RAW 264.7 cells pretreated with Compounds **5**, **18**, **20** and **21** have a similar effect at the same concentration. The inhibition activity was dose-dependent (Figure 3, Figure 4, Figure 5, Figure 6 and Figure 7).

The results suggested that the basal skeleton of guaiane-type sesquiterpene lactone (**18**, **20**, **21**) might be crucial for the inhibitory activity on NF-*κ*B production in RAW 264.7 cells. The functional groups such as hydroxyl groups linked at C-8 were found to decrease the inhibitory activity. The presence of the double bonds from the C-4 and C-11 contribution to the NF-*κ*B decreased the activity according the results of zaluzanin C (**14**) and dihydroestafiatol (**21**), while the glucosyl group at C-3 slightly rose the inhibitory activity on NF-*κ*B production. The significant inhibitory activity of gochnatiolide A (**5**) might be owed to the hydroxyl group linked at C-10 and the decrease of the methyl groups compared to other dimeric guaiane-type sesquiterpene lactones. The possible structure-activity relationships of these compounds should be further studied in the future, and it will be a significant topic.

## 3. Experimental Section

### 3.1. General Procedures

X-ray diffractions were measured on a Bruker SMART APEXII DUO (Bruker, Karlsruhe, Germany). Optical rotations were measured on a Perkin-Elmer 341 polarimeter (Perkin Elmer, Fremont, CA, USA). IR analyses were performed with a Nexus 470 FI-IR spectrometer (Thermo-Nicolet, Madison, WI, USA). UV spectra were recorded on Shimadzu UV/VIS-240 recording spectrophotometer (Shimadzu, Tokyo, Japan). 1D- and 2D-NMR spectra were obtained on a Bruker Avance 600 NMR spectrometer (Bruker, Karlsruhe, Germany). HR-ESIMS were acquired on an Agilent 6220 TOF LC-MS instrument (Agilent, Santa Clara, CA, USA). Column chromatography was performed by using silica gel (100–200 and 200–300 mesh; Yantai Jiangyou Silica Gel Development Co. Ltd., Yantai, China), Octadecylsilyl (50 μm; Wilmington, NC, USA), MCI Gel (75–150 μm; Mitsubishi Chemical Corporation, Tokyo, Japan); Sephadex LH-20 (40–70 μm; Pharmacia Company, Uppsala, Sweden). Semi-preparative HPLC isolation was achieved with an Agilent 1200 instrument (Agilent) equipped with a refractive index detector (RID), using a C_18_ column (250 mm × 10 mm × 5 μm, YMC) and eluting with ACN–H_2_O (30%–50%) and MeOH–H_2_O (40%–60%) at 2.0 mL/min. Precoated silica gel GF_254_ and HF_254_ plates were used for TLC, and zones were visualized under UV light (254 nm and 365 nm) or by spray with 10% H_2_SO_4_–EtOH followed by heating. 

### 3.2. Plant Material

The plant materials of *A. yunnanensis* were collected from Chuxiong, Yunnan province, during June 2013, and authenticated by Professor Ceming Tan (Jiujiang Forest Herbarium, Jiujiang, China). A voucher specimen (No. 20130629) was deposited at the Department of Pharmacognosy of the Second Military Medical University.

### 3.3. Extraction and Isolation

The air-dried *A. yunnanensis* (15 kg) was extracted three times with 80% ethanol (150 L) under reflux. After removal of the solvent by evaporation under vacuum, the residue was suspended in water (15 L) and then successively partitioned with petroleum, EtOAc and *n*-BuOH (3 × 15 L), respectively.

The EtOAc fraction (350 g) was chromatographed over a silica gel column (100–200 mesh, 120 × 1400 mm) and eluted with gradient CH_2_Cl_2_–MeOH (30:1 to 10:1) to obtain 5 fractions (A−E, respectively weighing 50 g, 63 g, 38 g, 16 g, 24 g). Fraction A was separated by silica gel column chromatography with CH_2_Cl_2_–MeOH (50:1 to 20:1) to obtain the fractions A1–A4 (17 g, 1 g, 7 g, 300 mg). Fraction A4 was separated on Sephadex LH-20 with CH_2_Cl_2_–MeOH (1:1) to yield Compound **12** (6 mg). Fraction B was separated on Sephadex LH-20 with CH_2_Cl_2_-MeOH (1:1) to obtain four subfractions (B1−B4); Fraction B2 (200 mg) was separated by silica gel column chromatography with CH_2_Cl_2_–MeOH (20:1 to 5:1) to obtain Compound **13** (19 mg) and the subfraction B2-1 (120 mg). The subfraction B2-1 was separated on Sephadex LH-20 with CH_2_Cl_2_–MeOH (1:1) to yield Compound **14** (75 mg). Fraction C (38 g) was separated on Sephadex LH-20 with CH_2_Cl_2_–MeOH (1:1) to obtain five subfractions (C1−C5); the fraction C4 (240 mg) was separated on the RP-18 column with MeOH–H_2_O (1:1–9:1) to yield Compounds **20** (8 mg) and **21** (10 mg). Fraction D was separated on Sephadex LH-20 with CH_2_Cl_2_–MeOH (1:1) to obtain the subfractions D1−D5 (3 g, 1.8 g, 4.2 g, 600 mg, 900 mg). The fraction D2 (1.8 g) was separated by HPLC on a semi-preparative C_18_ column with 48% ACN-H_2_O as the mobile phase to obtain Compound **5** (750 mg) (2.0 mL/min, 205 nm, *t*_R_ = 90.0 min) and five fractions (Fr.D2-1–D2-5). Fr.D2-1 (27 mg) was separated by reversed-phase HPLC eluting with 35% ACN-H_2_O to afford Compound **2** (13.0 mg) (2.0 mL/min, 205 nm, *t*_R_ = 49.0 min). Fr.D2-2 (253 mg) was separated by reversed-phase HPLC eluting with 38% ACN-H_2_O to afford Compound **6** (70 mg) (2.0 mL/min, 205 nm, *t*_R_ = 46.0 min), Compound **4** (4.6 mg) (2.0 mL/min, 205 nm, *t*_R_ = 52.0 min) and Compound **9** (28 mg) (2.0 mL/min, 205 nm, *t*_R_ = 58.0 min). Fr.D2-3 (162 mg) was separated by reversed-phase HPLC eluting with 40% ACN to afford Compound **1** (27 mg) (2.0 mL/min, 205 nm, *t*_R_ = 43.0 min). Fr.D2-4 (108 mg) was also separated by reversed-phase HPLC eluting with 45% ACN-H_2_O to afford Compound **3** (37 mg) (2.0 mL/min, 205 nm, *t*_R_ = 48.0 min). Fraction E (24 g) was subjected on the MCI gel column to MeOH–H_2_O (1:1–9:1) as the eluting solvent to afford five subfractions E1−E5 (6 g, 800 mg, 500 mg, 1.4 g, 7.2 g). Fraction E3 (500 mg) was subjected by column chromatography Sephadex LH-20 to CH_2_Cl_2_–MeOH (1:1) as the eluting solvent to afford four subfractions (E3-1–E3-4). Fraction E3-1 (26 mg) was separated by HPLC on a semi-preparative C_18_ column with 45% ACN-H_2_O respectively as the mobile phase to yield Compound **11** (9 mg) (2.0 mL/min, 205 nm, *t*_R_ = 43.0 min). Fraction E3-3 (72 mg) was separated by HPLC on a semi-preparative C_18_ column with 40% ACN-H_2_O respectively as the mobile phase to yield Compound **7** (4.5 mg) (2.0 mL/min, 205 nm, *t*_R_ = 49.0 min); Fraction E3-E4 (35 mg) were separated by HPLC on a semi-preparative C_18_ column with 38% ACN-H_2_O respectively as the mobile phase to yield Compound **8** (12 mg) (2.0 mL/min, 205 nm, *t*_R_ = 46.0 min) and Compound **10** (14 mg) (2.0 mL/min, 205 nm, *t*_R_ = 47.0 min). 

The n-BuOH fraction (400 g) was subjected to column chromatography on silica gel and gradient elution with CH_2_Cl_2_-MeOH (10:1 to 5:1) to obtain Fractions A–H (20 g, 19 g, 28 g, 33 g, 27 g, 15 g, 30 g, 68 g). The fraction D was separated on Sephadex LH-20 with CH_2_Cl_2_–MeOH (1:1) to obtain three subfractions D1–D3 (8 g, 1.2 g, 11 g); the fraction D2 (1.2 g) was separated on the ODS gel column chromatography with MeOH–H_2_O (1:4 to 5:1) to obtain Compound **17** (12 mg) and Subfraction D2-1 (70 mg); then, the subfraction D2-1 was separated on Sephadex LH-20 with CH_2_Cl_2_-MeOH (1:1) to obtain Compound **15** (6 mg). Fraction F (15 g) was separated on Sephadex LH-20 with CH_2_Cl_2_-MeOH (1:1) to obtain four subfractions F1−F4 (2 g, 900 mg, 600 mg, 7 g). Fraction F2 (900 mg) was separated by MCI gel column chromatography with MeOH–H_2_O (1:4 to 4:1) to obtain Compound **16** (5 mg). Fraction F3 (600 mg) was separated by ODS gel column chromatography with MeOH–H_2_O (1:4–4:1) to obtain Compound **18** (200 mg) and the subfraction F3-1 (80 mg), then the fraction F3-1 was separated by HPLC on a semi-preparative C_18_ column (250 mm × 10 mm, 2.0 mL/min.) with 30% ACN-H_2_O as the mobile phase to afford the Compound **19** (16 mg).

### 3.4. Luciferase Assay

The NF-*κ*B 293 cells were cultured in DMEM supplemented with 10% fetal bovine serum (FBS). The cells were pretreated with Compounds **1**–**21** at concentrations of 1, 10, 100 μg/mL for 4 h and then stimulated with 10 μg/mL lipopolysaccharide (LPS) for 24 h. The cells were rinsed twice with phosphate-buffered saline (PBS, pH 7.4) and lysed with passive lysis buffer (Promega, Madison, WI, USA). Then, the inhibitory effect on NF-*κ*B was analyzed using the luciferase assay system (Promega) according to the manufacturer′s instructions [26].

### 3.5. Measurement of TNF-α, IL-1β, IL-6 and IL-10

The cells were cultured in serum-free medium for 8 h and then incubated in medium containing 1, 10, 100 μg/mL of Compounds **5**, **18**, **20** and **21** for 2 h. The cells were then treated with 10 μg/mL of LPS for 24 h. Ibuprofen (1, 10, 100 μg/mL) was used as a positive control. The supernatants of the cell culture were harvested and centrifuged at 3000× *g* at 4 °C for 2 min for the analysis of TNF-*α*, IL-1β, IL-6 and IL-10. Enzyme-linked immunosorbent assays for detecting the cytokines in the supernatants were carried out according to the instructions provided by the manufacturer. Finally, the standard provided with the kits was used to quantify each cytokine in the supernatants [27].

### 3.6. Characterization of Compounds

Ainsliadimer F (**1**): colorless, prisms; UV (MeOH) (log ε) λ_max_ 205 (1.84); [*α*${]}_{D}^{20}$−60.4 (*c* 0.60, MeOH); IR (KBr) ν_max_ 3458, 3075, 2931, 2875, 2025, 1774, 1700, 1639, 1456, 1385, 1338, 1311, 1260, 1218, 1183, 1141, 1036, 999, 963, 936, 919, 874, 850, 813 cm^−1^; ^1^H- and ^13^C-NMR data; see Table 1; HRESIMS *m*/*z*: 529.2206 [M + Na]^+^ (calcd. for C_30_H_34_O_7_, 506.2110).

Ainsliadimer G (**2**): white, amorphous powder; UV (MeOH) (log ε) λ_max_ 204 (1.42); [*α*${]}_{D}^{20}$−92.6 (*c* 0.37, MeOH); IR (KBr) ν_max_ 3491, 3075, 2925, 2851, 2025, 1764, 1735, 1699, 1638, 1447, 1409, 1339, 1311, 1265, 1129, 1080, 1042, 1001, 968, 915, 874, 814, 735 cm^−1^; ^1^H- and ^13^C-NMR data; see Table 1; HRESIMS *m*/*z*: 557.2148 [M + Na]^+^ (calcd. for C_31_H_34_O_8_, 534.2052).

Ainsliadimer H (**3**): white, amorphous powder; UV (MeOH) (log ε) λ_max_ 204 (1.40); [*α*${]}_{D}^{20}$−157.0 (*c* 0.10, MeOH); IR (KBr) ν_max_ 3490, 3076, 2927, 2868, 2025, 1765, 1736, 1699, 1639, 1446, 1408, 1338, 1310, 1264, 1216, 1129, 1042, 1001, 972, 916, 814 cm^−1^; ^1^H- and ^13^C-NMR data; see Table 1; HRESIMS *m*/*z*: 571.2310 [M + Na]^+^, (calcd. for C_32_H_36_O_8_, 548.2214).

Ainsliadimer I (**4**): white, amorphous powder; UV (MeOH) (log ε) λ_max_ 204 (1.35); [*α*${]}_{D}^{20}$−88.5 (*c* 0.13, MeOH); IR (KBr) ν_max_ 3447, 2924, 2852, 2025, 1769, 1699, 1445, 1404, 1385, 1307, 1259, 1212, 1138, 1038, 998, 947, 813 cm^−1^; ^1^H- and ^13^C-NMR data; see Table 1; HRESIMS *m*/*z* 527.2043 [M + Na]^+^ (calcd. for C_30_H_32_O_7_, 504.1947). 

X-ray crystallographic study of ainsliadimer F (**1**): Crystal data: λ = 1.54178 Α, T = 193 (2) K, space group P21, 21, 21, a = 8.7146 (4), b = 15.2221 (6), c = 20.7579 (8). V = 2753.6 (2) A^3^, Z = 4, Dx = 1.309 g/cm^3^, crystal size: 0.65 × 0.19 × 0.17 mm^3^.

## 4. Conclusions

Four new dimeric sesquiterpene lactones, ainsliadimer F–I (**1**–**4**), together with seven known dimeric sesquiterpene lactones (**5**–**11**) and ten sesquiterpenes (**12**–**21**) have been obtained from *A. yunnanensis*. All of these compounds were reported to be isolated from *A. yunnanensis* for the first time. Compounds **5**, **18**, **20** and **21** showed significant activities in anti-inflammatory assays. Additionally, they exhibited good anti-inflammatory effects in a dose-dependent manner. The observed potential anti-inflammatory activity warrants further investigations. 

## Figures and Tables

**Table 1:** ^1^H- (600 MHz) and ^13^C- (150 MHz) NMR spectroscopic data of Compounds **1**–**4** ^a^.

No.	1		2		3		4	
	^1^H	^13^C	^1^H	^13^C	^1^H	^13^C	^1^H	^13^C
1	-	172.0, qC	-	173.2, qC	-	173.1, qC	-	171.6, qC
2	-	140.2, qC	-	140.6, qC	-	140.8, qC	-	140.4, qC
3	-	208.5, qC	-	206.3, qC	-	206.5, qC	-	208.4, qC
4	2.52 (dd, 7.2, 4.2)	47.0, CH	2.63, m	52.0, CH	2.62, m	51.8, CH	2.56 (dd, 7.1, 4.2)	47.0, CH
5	2.80 (dd, 11.1, 4.1)	54.8, CH	3.52 (dd, 12.3, 5.3)	48.3, CH	3.52 (dd, 11.3, 4.1)	48.5, CH	2.91 (dd, 11.2, 4.2)	55.1, CH
6	4.32 (dd, 11.0, 9.6)	82.6, CH	4.38 (dd, 11.2, 9.8)	82.6, CH	4.36 (dd, 11.3, 9.7)	82.6, CH	4.33 (dd, 11.1, 9.7)	82.6, CH
7	1.84, m	54.9, CH	2.87, m	51.6, CH	2.86, m	51.7, CH	2.75, m	51.6, CH
8	2.05, m 1.85, m	22.5, CH_2_	2.16, m 2.08, m	21.0, CH_2_	2.11, m 2.03, m	21.0, CH_2_	2.10, m 2.02, m	21.0, CH_2_
9	1.97, m 1.70, m	36.2, CH_2_	2.00, m 1.73, m	36.2, CH_2_	1.95, m 1.73, m	36.2, CH_2_	1.71, m 1.68, m	36.1, CH_2_
10	-	68.1, qC	-	68.4, qC	-	68.4, qC	-	68.3, qC
11	2.35, m	41.9, CH	-	139.5, qC	-	139.6, qC	-	139.4, qC
12	-	178.6, qC	-	170.1, qC	-	170.1, qC	-	170.0, qC
13	1.27 (d, 6.9)	12.6, CH_3_	6.23 (d, 3.3) 5.56 (d, 3.0)	119.1, CH_2_	6.21 (d, 3.3) 5.54 (d, 3.1)	119.1, CH_2_	6.20 (d, 3.2) 5.52 (d, 3.1)	119.1, CH_2_
14	1.86, m 1.69, m	36.3, CH_2_	2.12, m 1.94, m	36.0, CH_2_	2.09, m 1.93, m	36.0, CH_2_	1.86, m 1.69, m	36.3, CH_2_
15	1.27 (d, 7.1) -	14.3, CH_3_	3.94 (dd, 9.5, 2.2) 3.74 (dt, 9.5, 3.2)	69.2, CH_2_	3.96 (dd, 9.6, 2.3) 3.76 (dd, 9.7, 3.1)	67.2, CH_2_	1.30 (d, 7.2) -	14.2, CH_3_
1′	3.22, m	40.0, CH	3.22, m	40.0, CH	3.24 (t, 9.3)	40.0, CH	3.22 (t, 9.0)	40.0, CH
2′	3.22, m 2.63, m	44.7, CH_2_	3.22, m 2.63, m	44.8, CH_2_	2.68, m 2.62, m	44.8, CH_2_	3.23, m 2.64, m	44.7, CH_2_
3′	-	222.3, qC	-	222.5, qC	-	222.4, qC	-	222.1, qC
4′	-	50.9, qC	-	50.9, qC	-	50.9, qC	-	50.9, qC
5′	3.20 (d, 4.9)	49.6, CH	3.25 (t, 10.2)	49.6, CH	3.17 (t, 9.3)	49.7, CH	3.19 (t, 8.7)	49.5, CH
6′	4.16 (t, 9.4)	83.9, CH	4.19 (t, 9.2)	83.9, CH	4.18 (t, 9.8)	83.8, CH	4.17 (t, 9.2)	83.9, CH
7′	3.00, m	43.5, CH	3.05, m	43.4, CH	3.03, m	43.5, CH	3.01, m	43.5, CH
8′	2.31, m 1.47, m	31.9, CH_2_	2.35, m 1.51, m	32.0, CH_2_	2.33, m 1.51, m	32.0, CH_2_	2.35, m 1.48, m	31.9, CH_2_
9′	2.64, m 2.20, m	39.5, CH_2_	2.65, m 2.24, m	39.5, CH_2_	2.64, m 2.23, m	39.5, CH_2_	2.65, m 2.22, m	39.5, CH_2_
10′	-	150.1, qC	-	150.2, qC	-	150.2, qC	-	150.1, qC
11′	-	138.5, qC	-	138.6, qC	-	138.5, qC	-	138.4, qC
12′	-	169.3, qC	-	169.4, qC	-	169.2, qC	-	169.3, qC
13′	6.26 (d, 3.4) 5.58 (d, 3.0)	121.6, CH_2_	6.29 (d, 3.4) 5.60 (d, 3.0)	121.5, CH_2_	6.27 (d, 3.4) 5.59 (d, 3.0)	121.5, CH_2_	6.27 (d, 3.4) 5.58 (d, 3.0)	121.7, CH_2_
14′	5.09, brs 4.72, brs	114.1, CH_2_	5.12, brs 4.75, brs	114.1, CH_2_	5.11, brs 4.74, brs	114.1, CH_2_	5.09, brs 4.72, brs	114.1, CH_2_
15′	2.13, m 2.02, m	25.8, CH_2_	2.12, m 2.07, m	25.9, CH_2_	2.12, m 2.07, m	25.9, CH_2_	2.11, m 1.97, m	25.8, CH_2_
1″	-	-	3.29, s	59.2, OCH_3_	3.42 (dd, 7.0, 3.5)	66.8, OCH_2_	-	-
2″	-	-	-	-	1.06 (t, 7.0)	15.0, CH_3_	-	-

^a^ **1**–**4** was measured in CDCl_3_.

## Supplementary Materials

Supplementary materials can be accessed at: http://www.mdpi.com/1420-3049/21/8/1031/s1.
