# Supplementary material for: New Sesquiterpenenoids from Ainsliaea yunnanensis
Source: Molecules. 2016 Aug 8;21(8):1031. doi: 10.3390/molecules21081031 (PMC6273759; doi:10.3390/molecules21081031)
Supplement: Supplementary file 1 [file molecules-21-01031-s001.pdf]

Supplementary Materials: New Sesquiterpenenoids from *Ainsliaea yunnanensis*

Xiang-Lei Wu, Xiao-Juan Xiong, Wen-Quan Lu, Hao Huang, Yun-Heng Shen, Zhi-Jun Wu and Wan-Sheng Chen

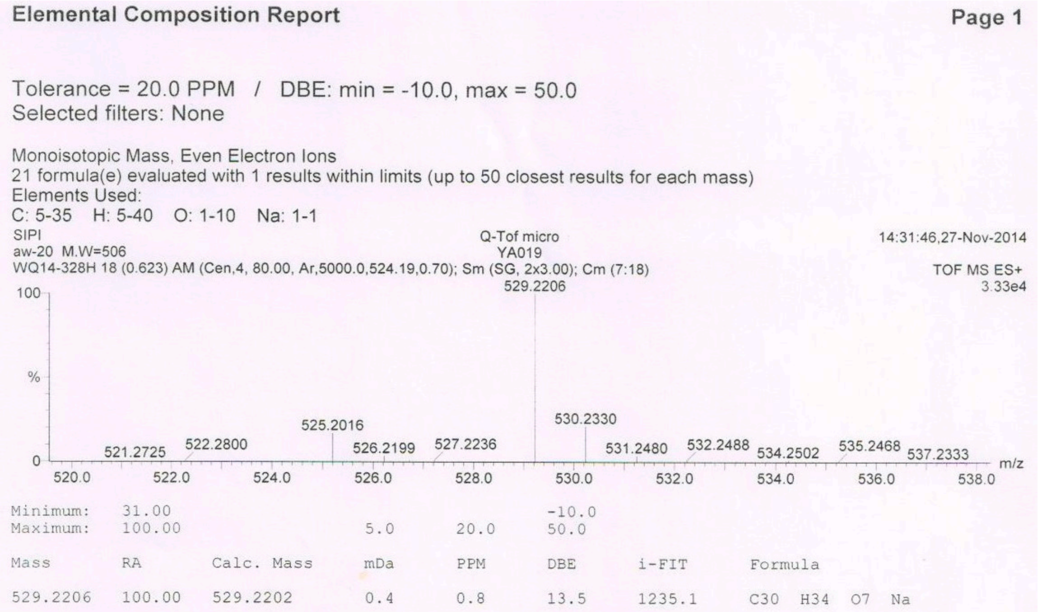

Figure S1. HRESI mass spectrum of compound **1**.

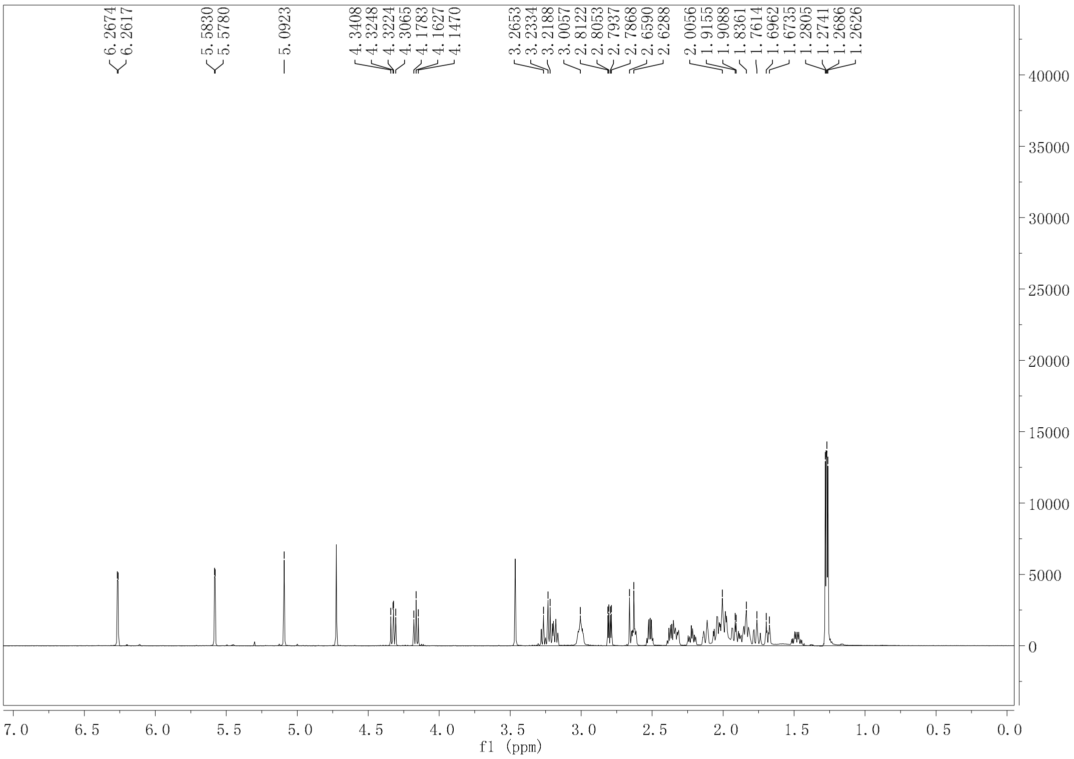

Figure S2. ^{1}H-NMR spectrum of compound **1** (600 MHz, in $CDCl_3$).

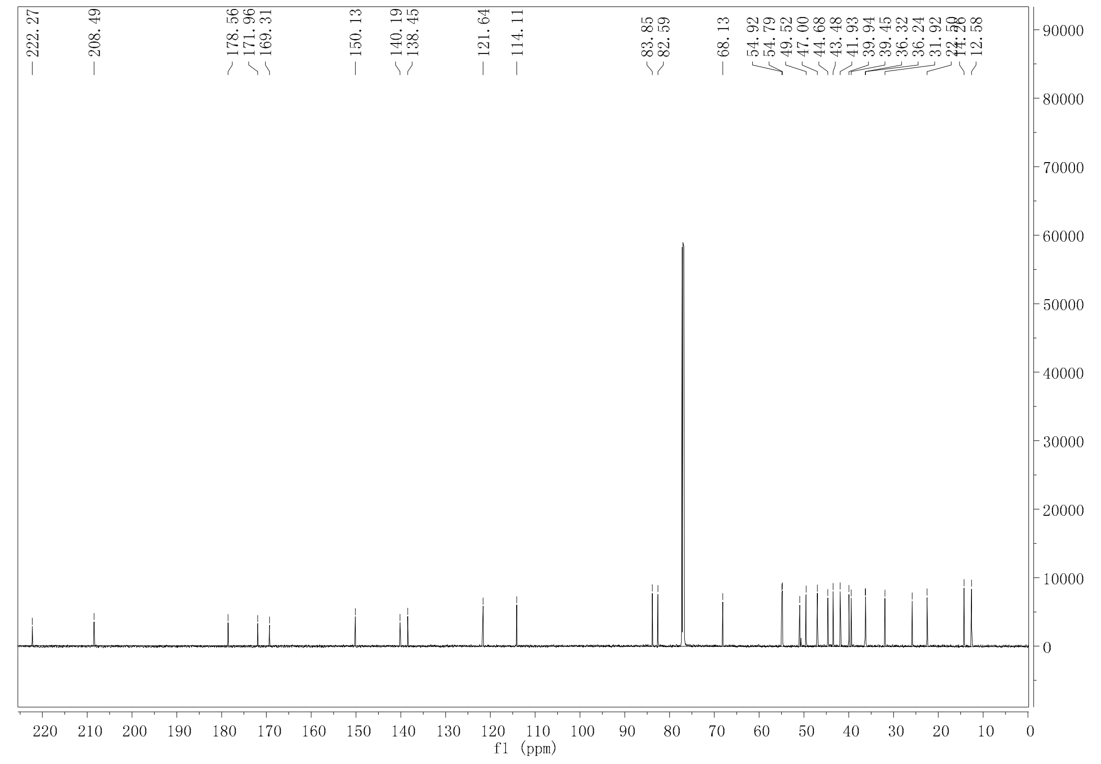

Figure S3. ^{13}C-NMR spectrum of compound **1** (150 MHz, in $CDCl_3$).

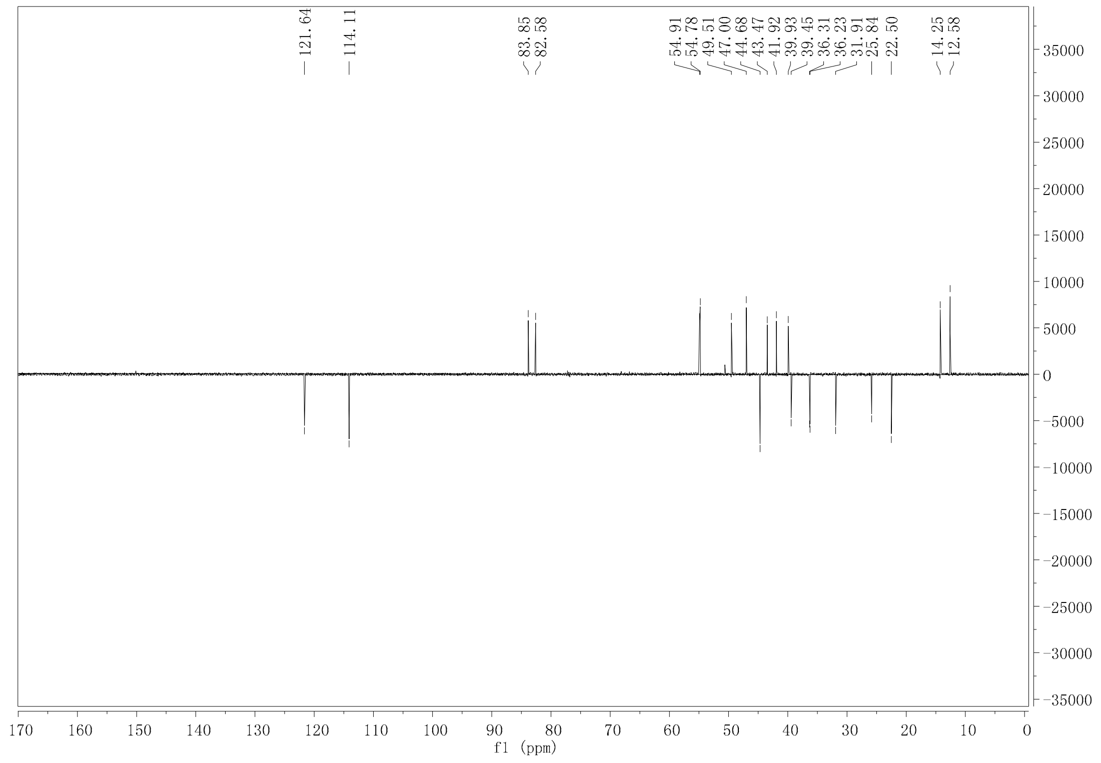

Figure S4. DEPT spectrum of compound **1** (150 MHz, in $CDCl_3$).

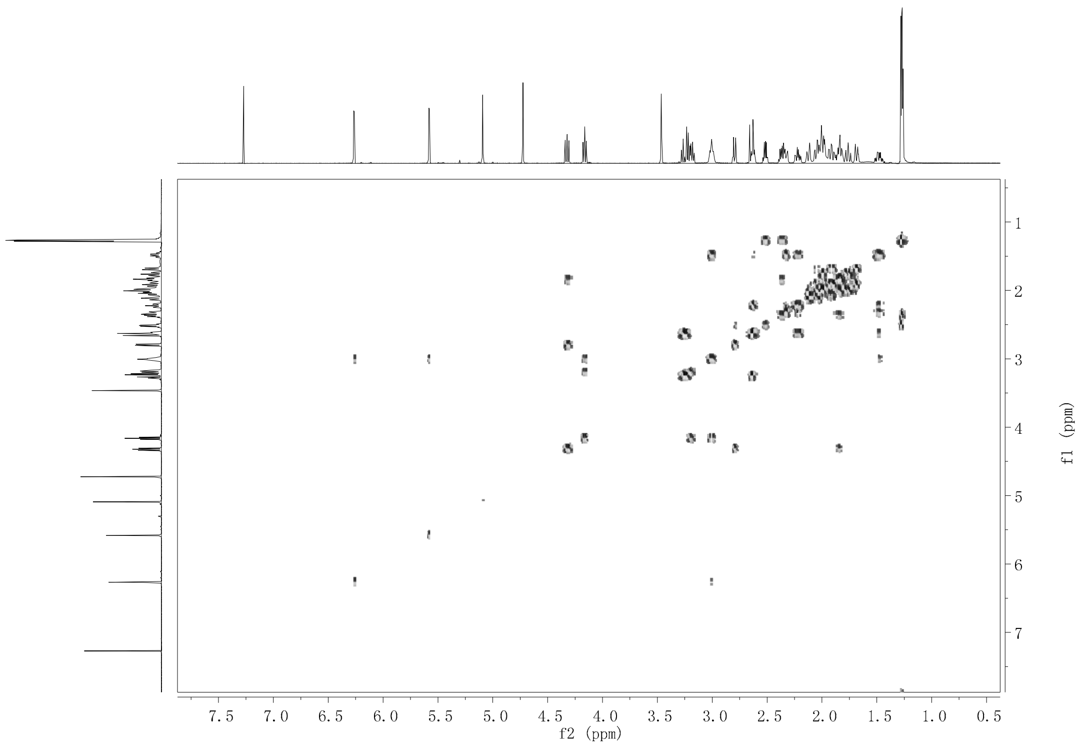

Figure S5. ^{1}H-^{1}H COSY spectrum of compound **1** (600 MHz, in $CDCl_3$).

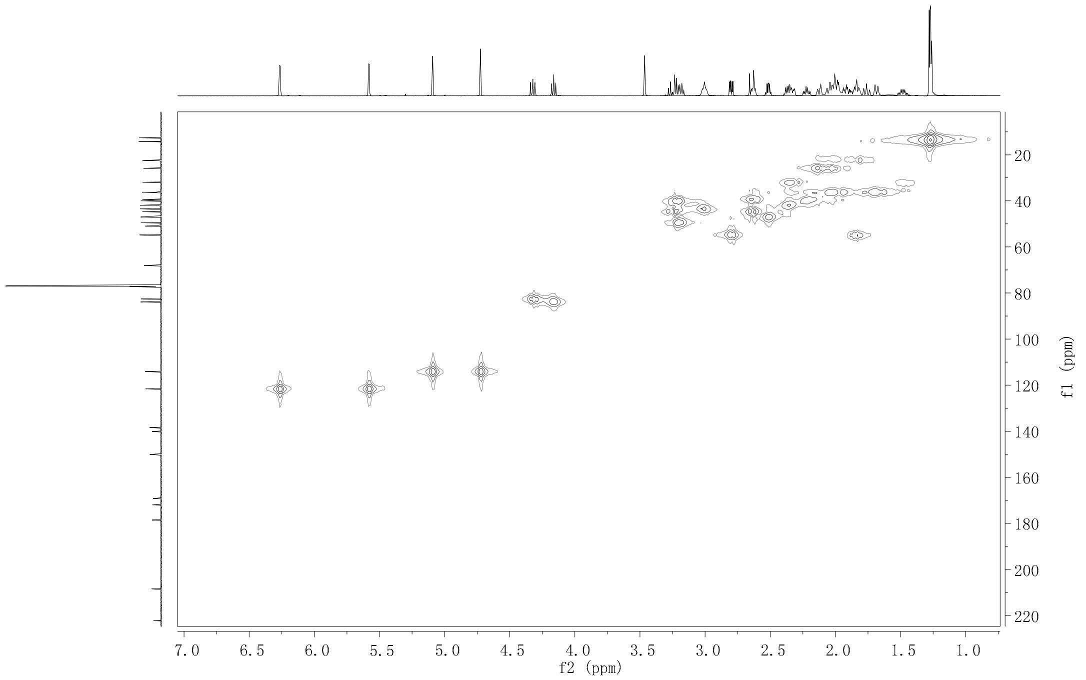

Figure S6. HSQC spectrum of compound **1** (600 MHz, in $CDCl_3$).

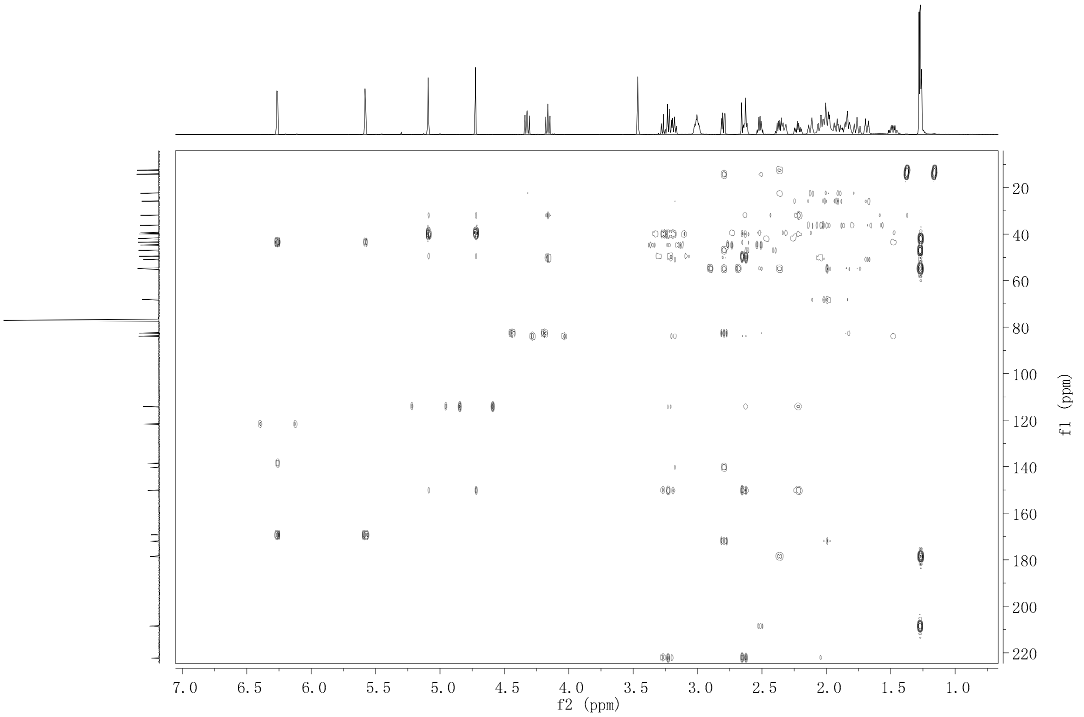

Figure S7. HMBC spectrum of compound **1** (600 MHz, in $CDCl_3$).

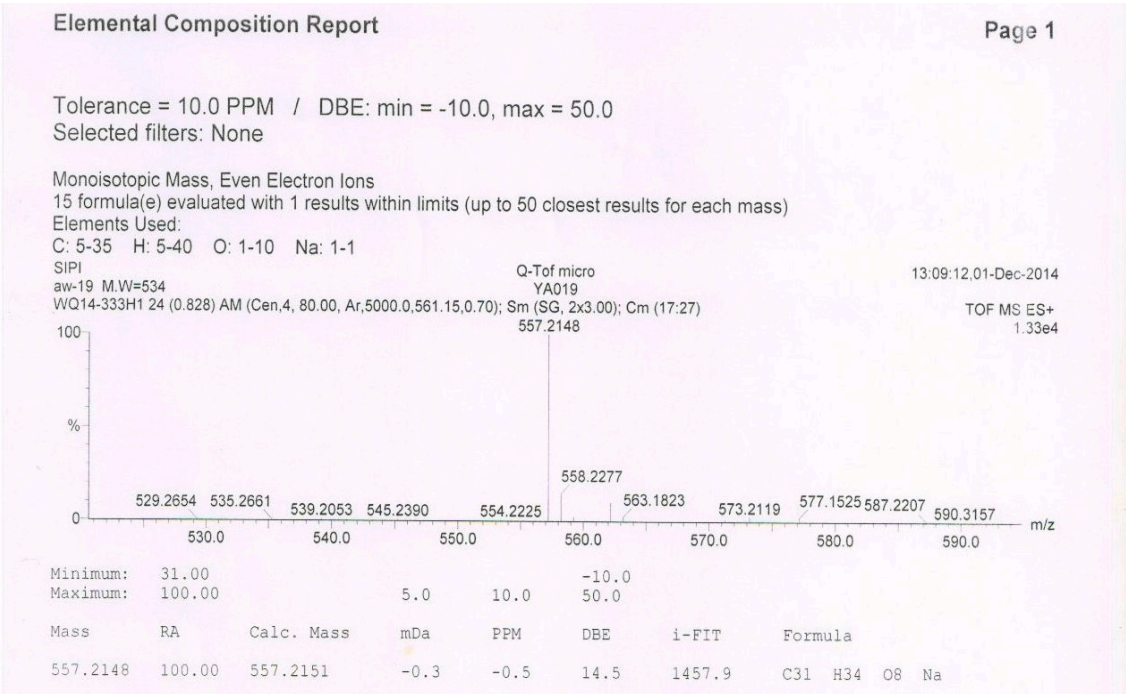

Figure S8. HRESI mass spectrum of compound **2**.

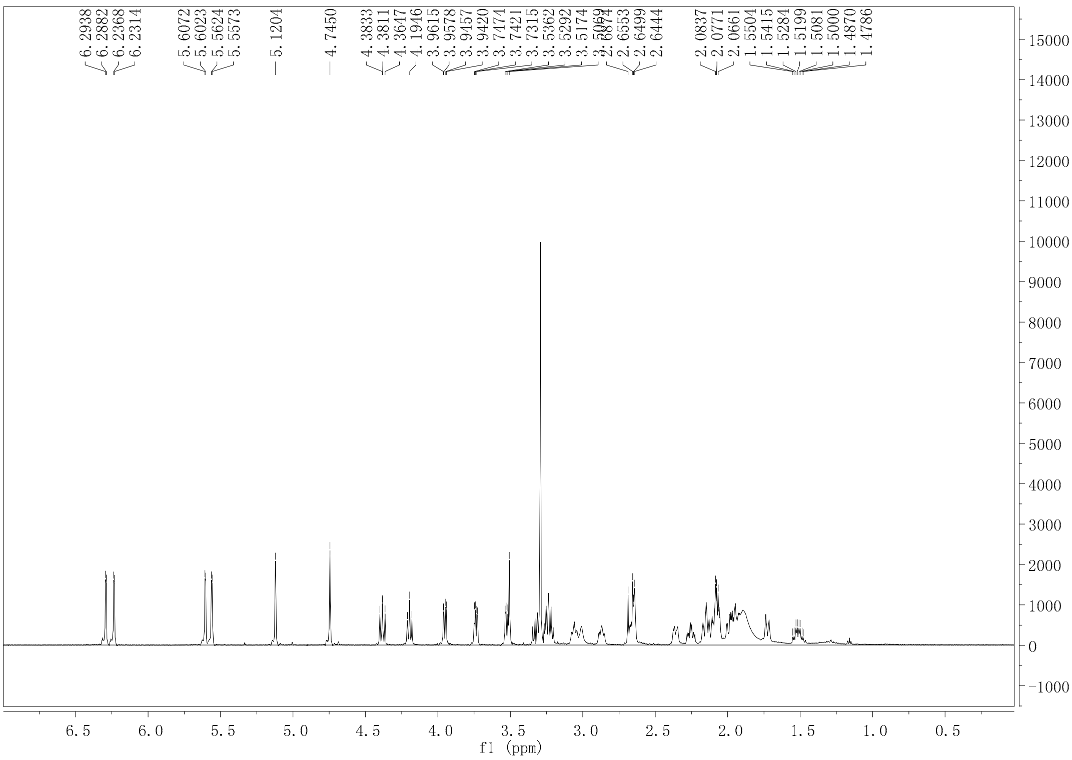

Figure S9. ^{1}H-NMR spectrum of compound **2** (600 MHz, in $CDCl_3$).

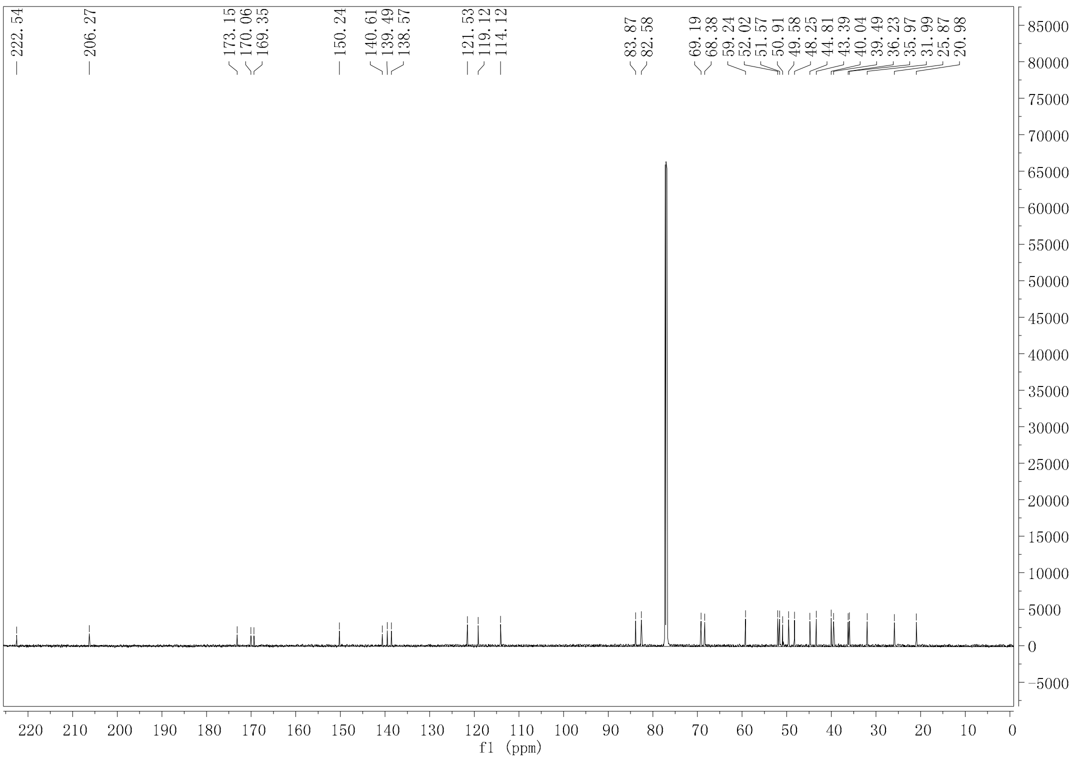

Figure S10. ^{13}C-NMR spectrum of compound **2** (150 MHz, in $CDCl_3$).

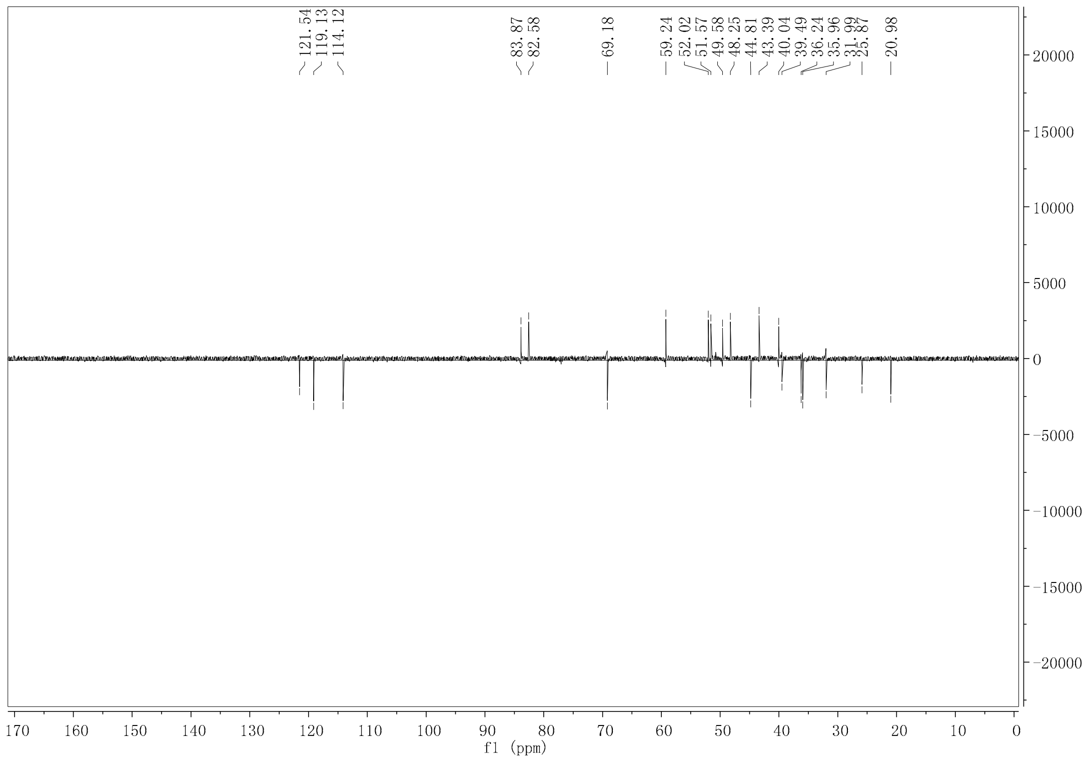

Figure S11. DEPT spectrum of compound **2** (150 MHz, in $CDCl_3$).

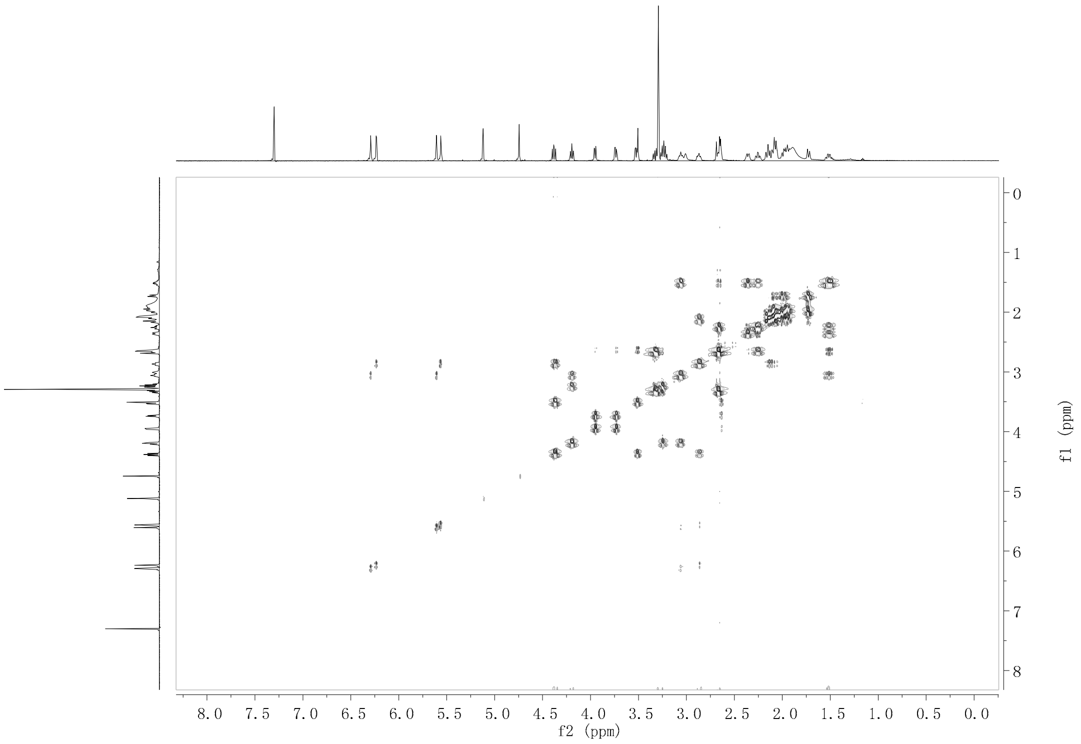

Figure S12. ^1H-^1H COSY spectrum of compound **2** (600 MHz, in $CDCl_3$).

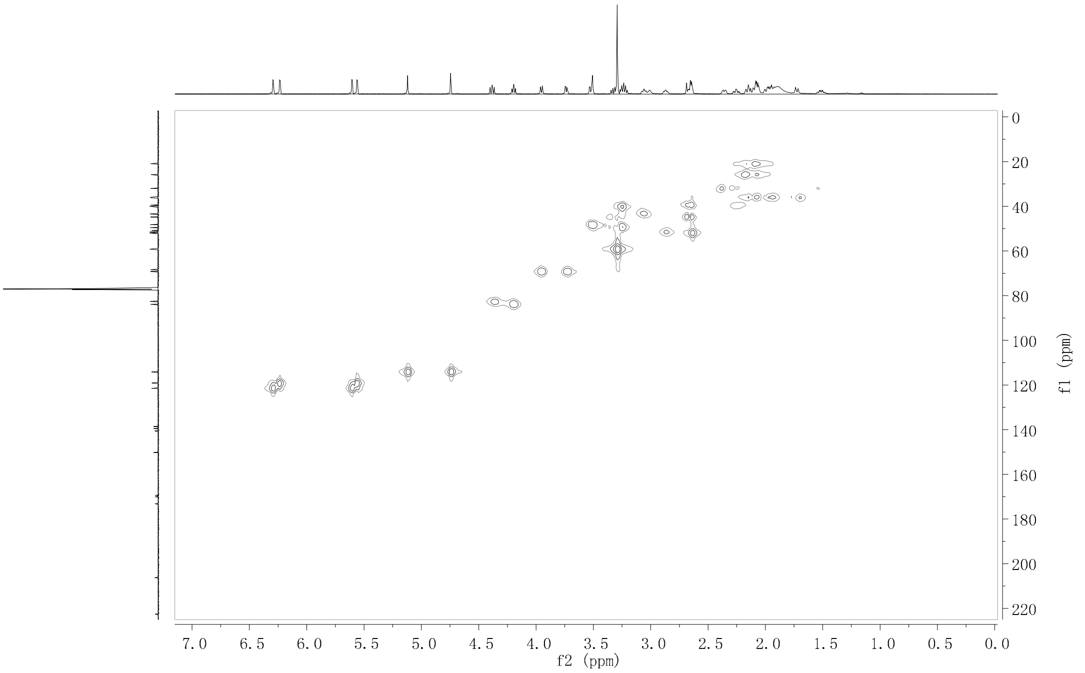

Figure S13. HSQC spectrum of compound **2** (600 MHz, in $CDCl_3$).

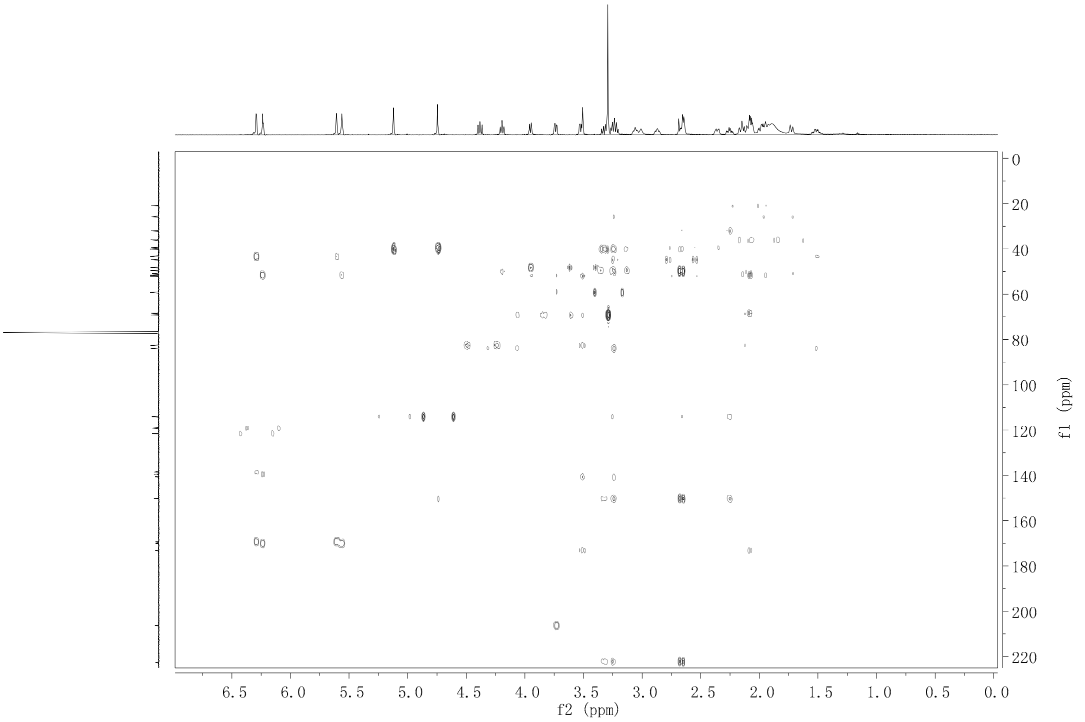

Figure S14. HMBC spectrum of compound **2** (600 MHz, in $CDCl_3$).

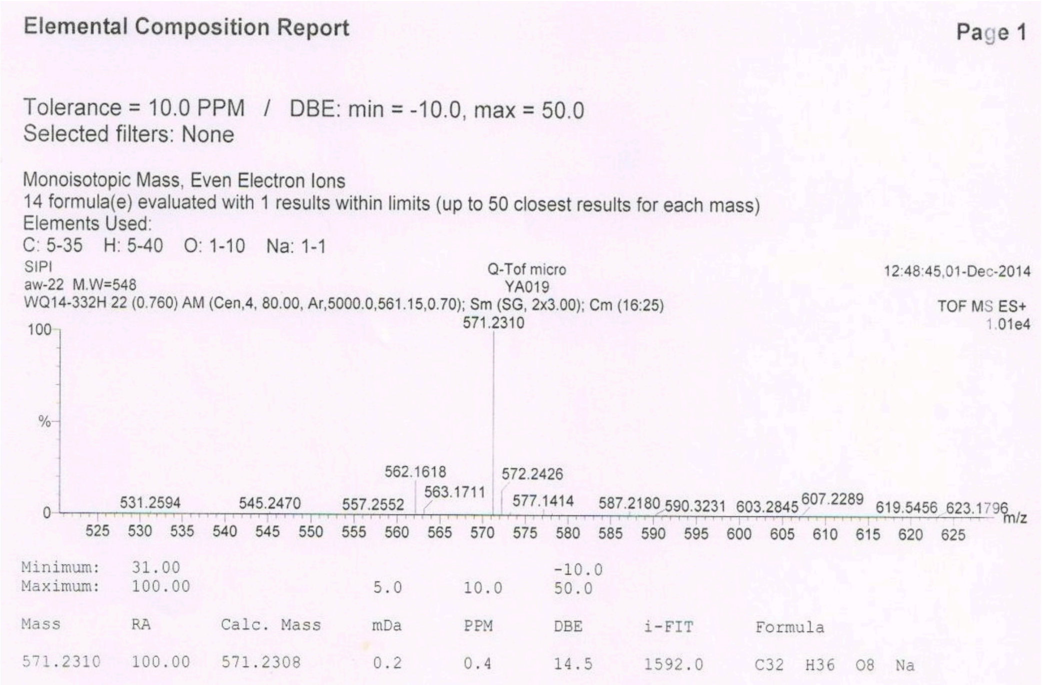

Figure S15. HRESI mass spectrum of compound **3**.

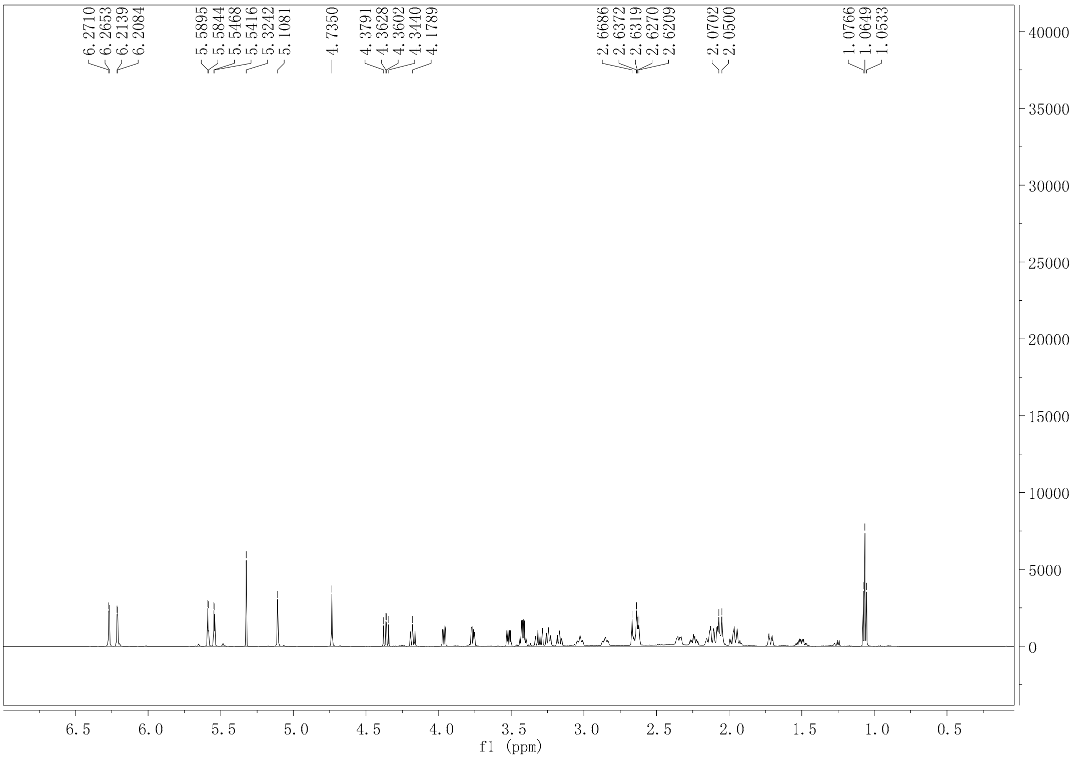

Figure S16. ^{1}H-NMR spectrum of compound **3** (600 MHz, in $CDCl_3$).

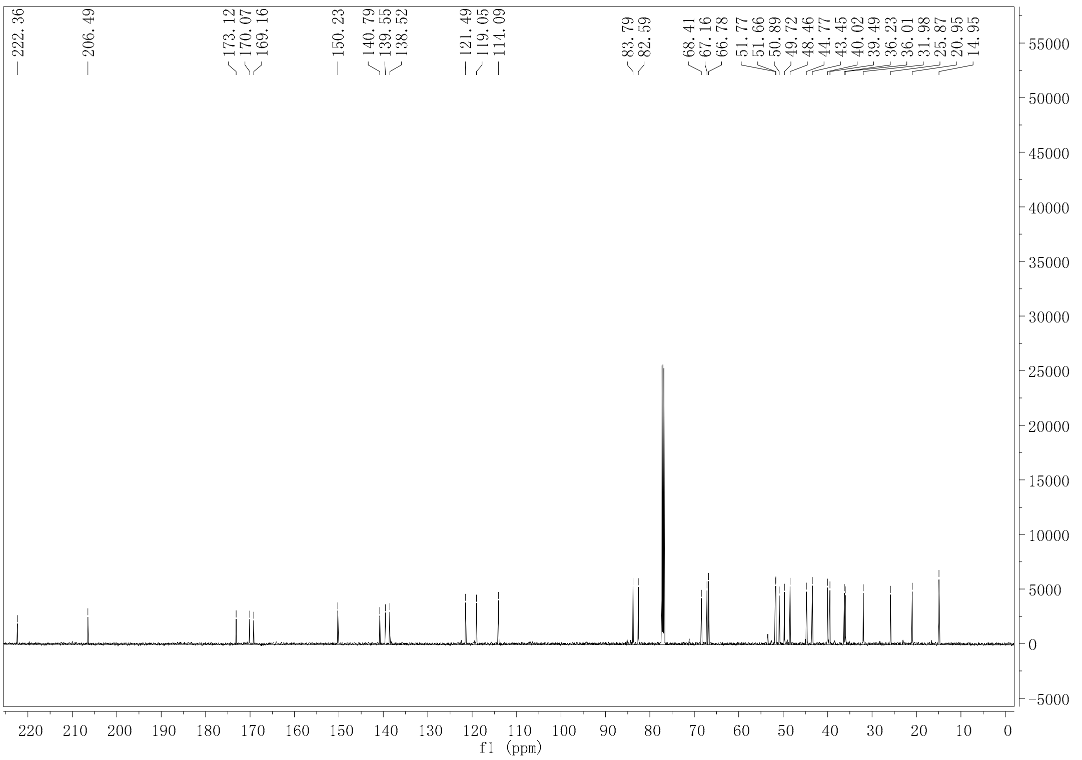

Figure S17. ^{13}C-NMR spectrum of compound **3** (150 MHz, in $CDCl_3$).

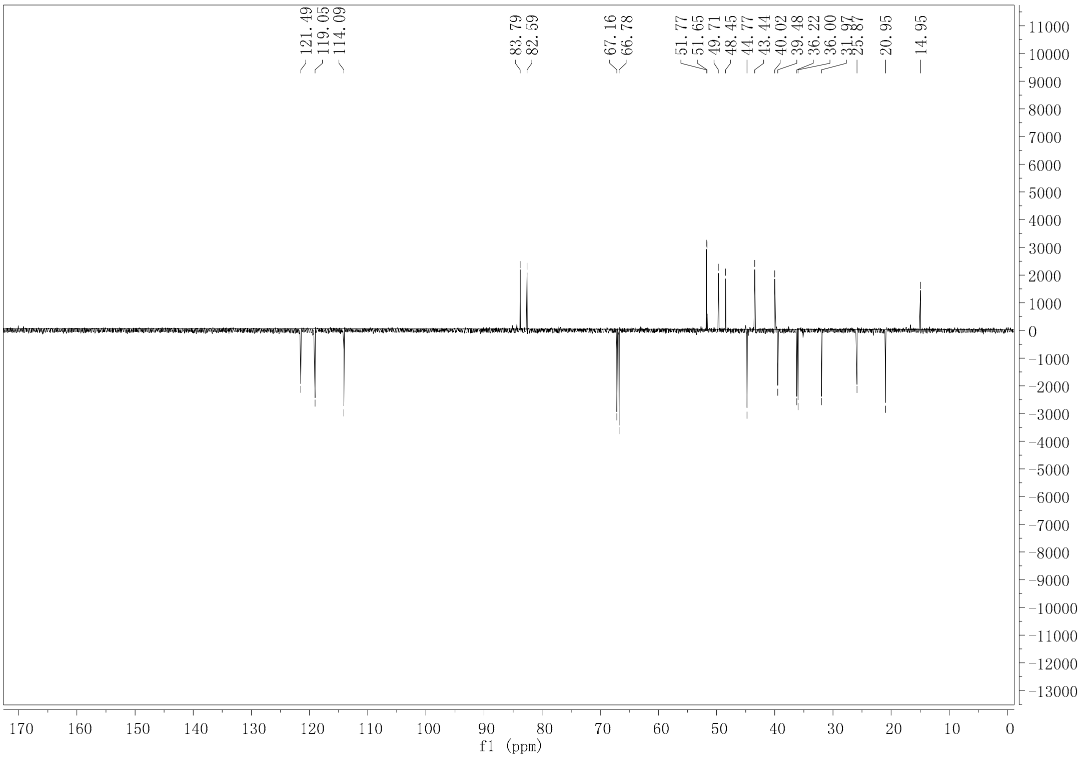

Figure S18. DEPT spectrum of compound **3** (150 MHz, in $CDCl_3$).

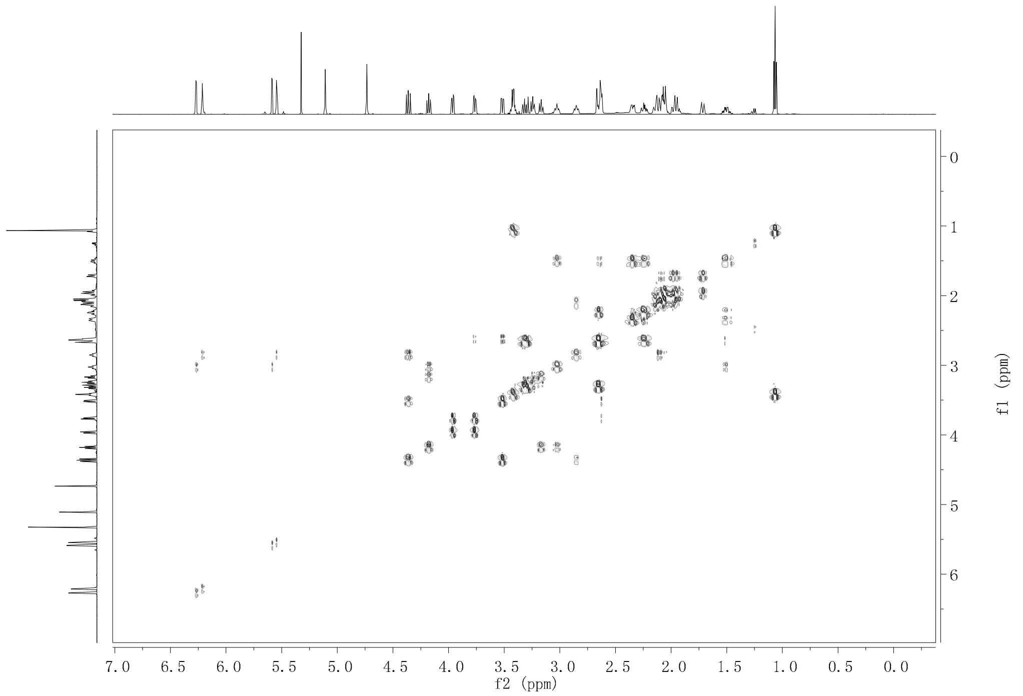

Figure S19. ^{1}H-^{1}H COSY spectrum of compound **3** (600 MHz, in $CDCl_3$).

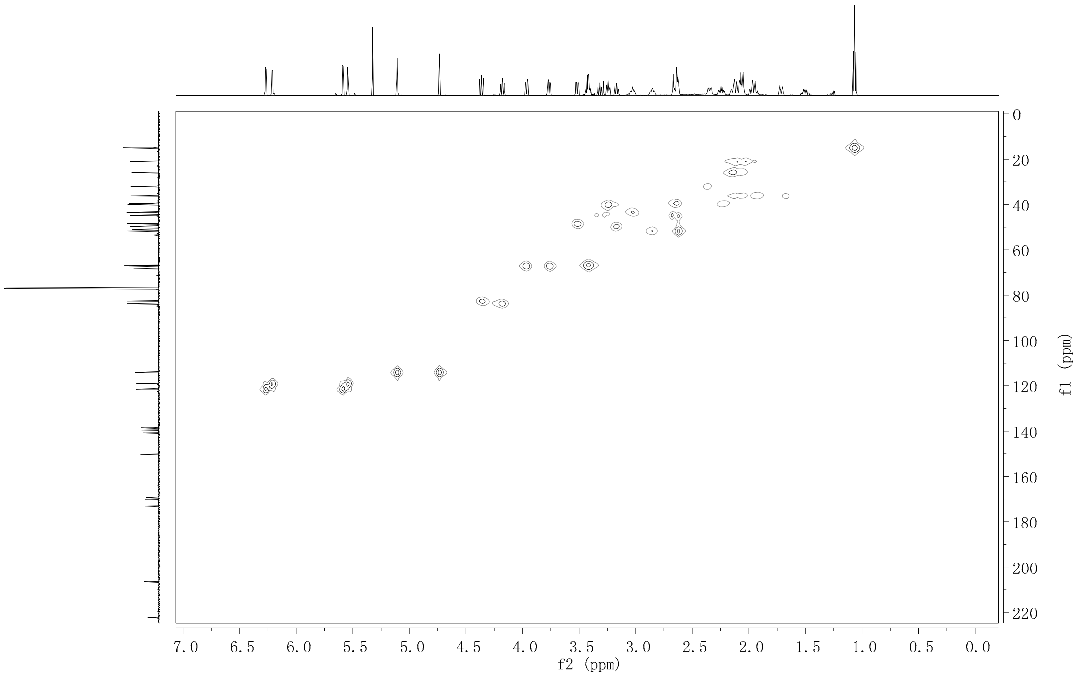

Figure S20. HSQC spectrum of compound **3** (600 MHz, in $CDCl_3$).

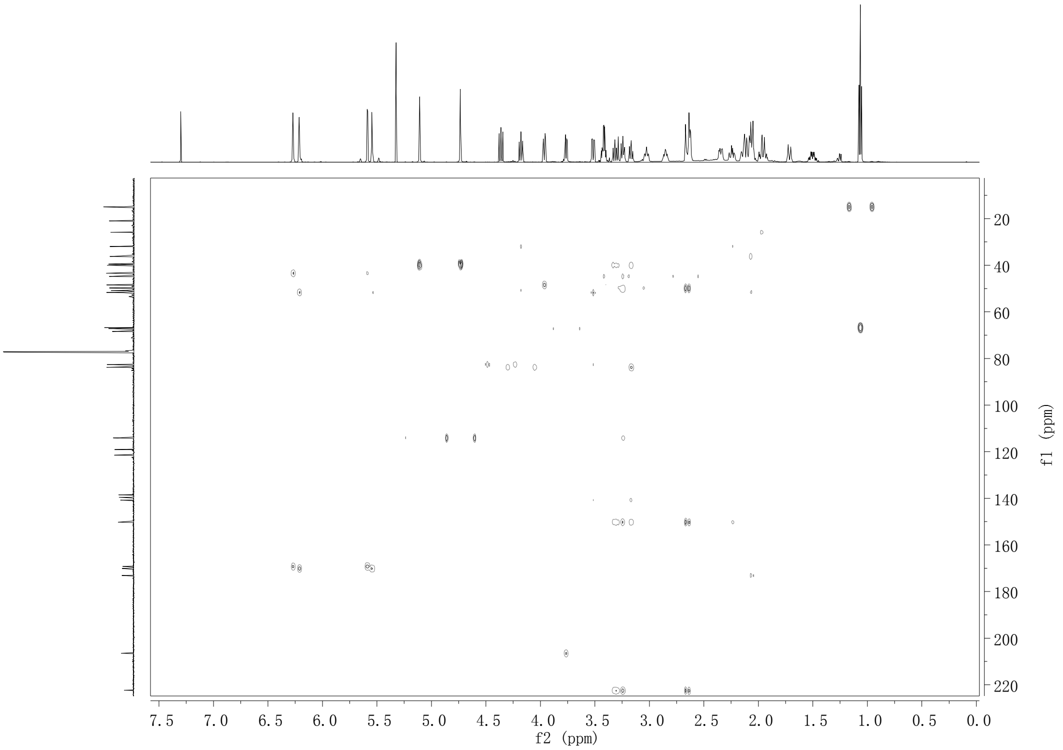

Figure S21. HMBC spectrum of compound **3** (600 MHz, in $CDCl_3$).

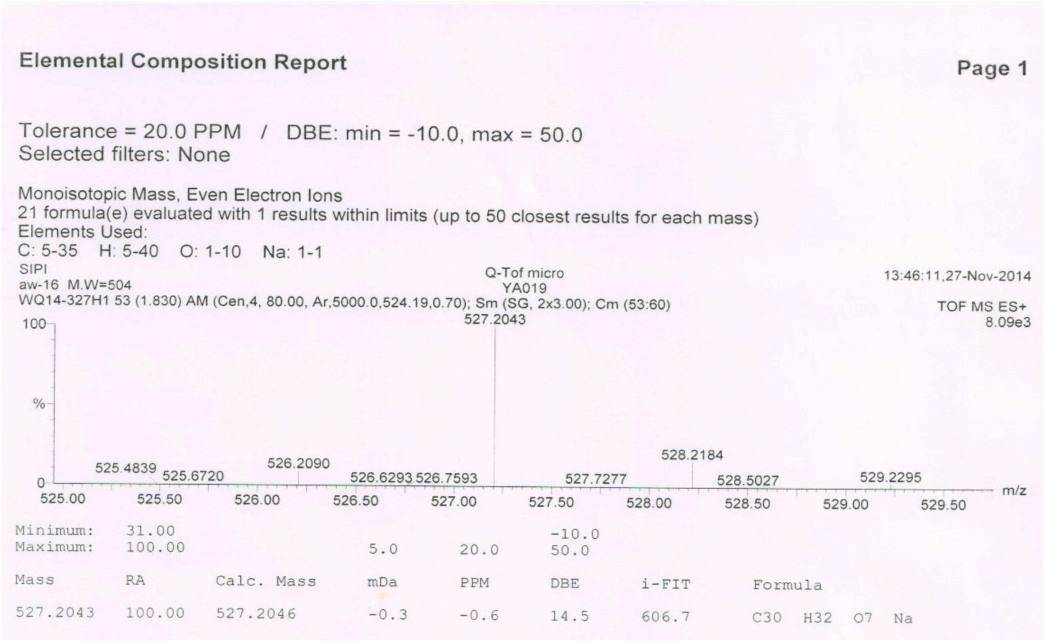

Figure S22. HRESI mass spectrum of compound **4**.

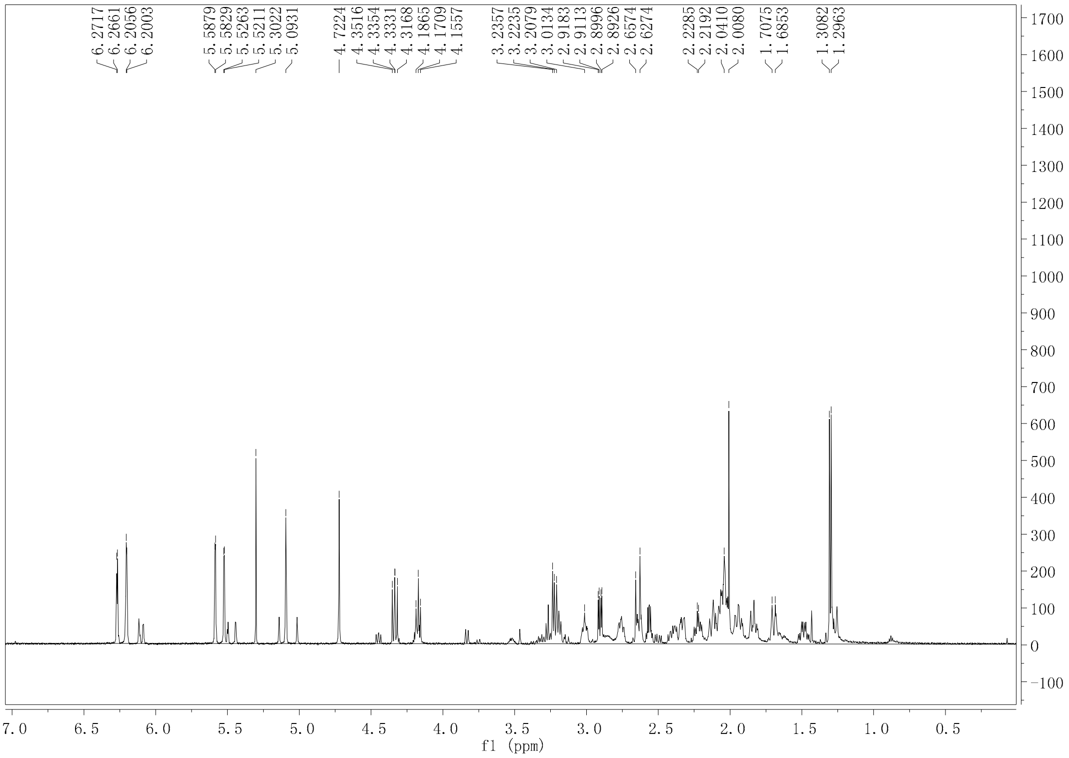

Figure S23. ^{1}H-NMR spectrum of compound **4** (600 MHz, in CDCl3).

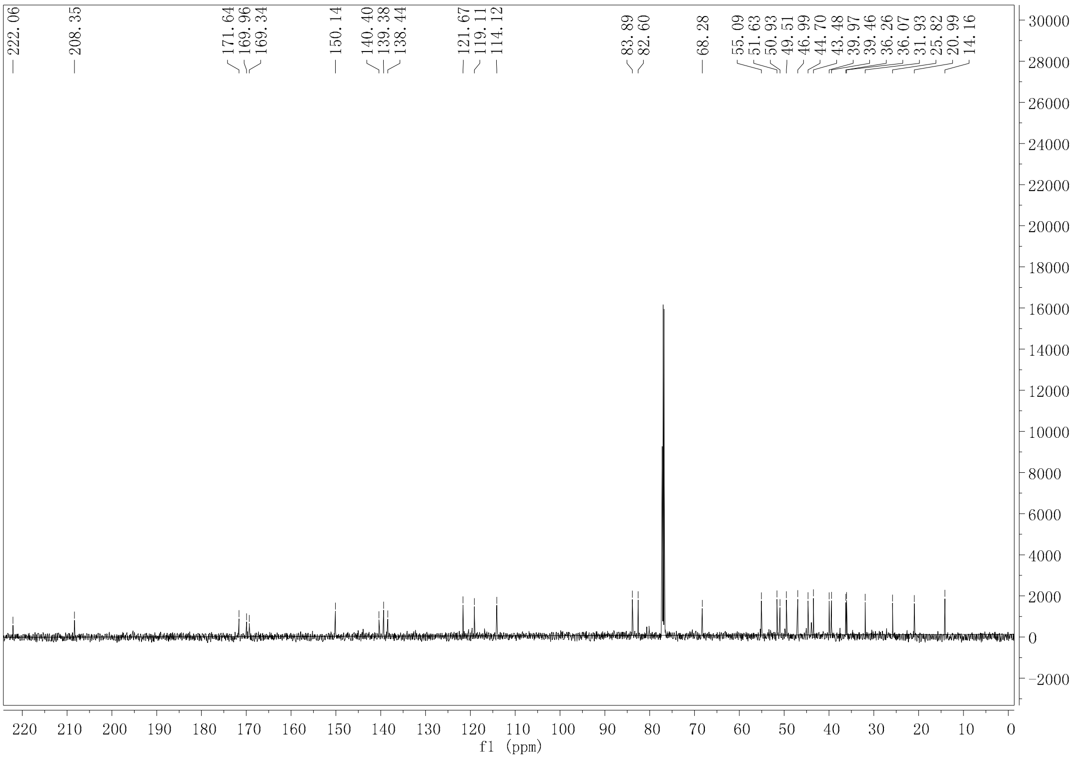

Figure S24. ^{13}C-NMR spectrum of compound **4** (150 MHz, in $CDCl_3$).

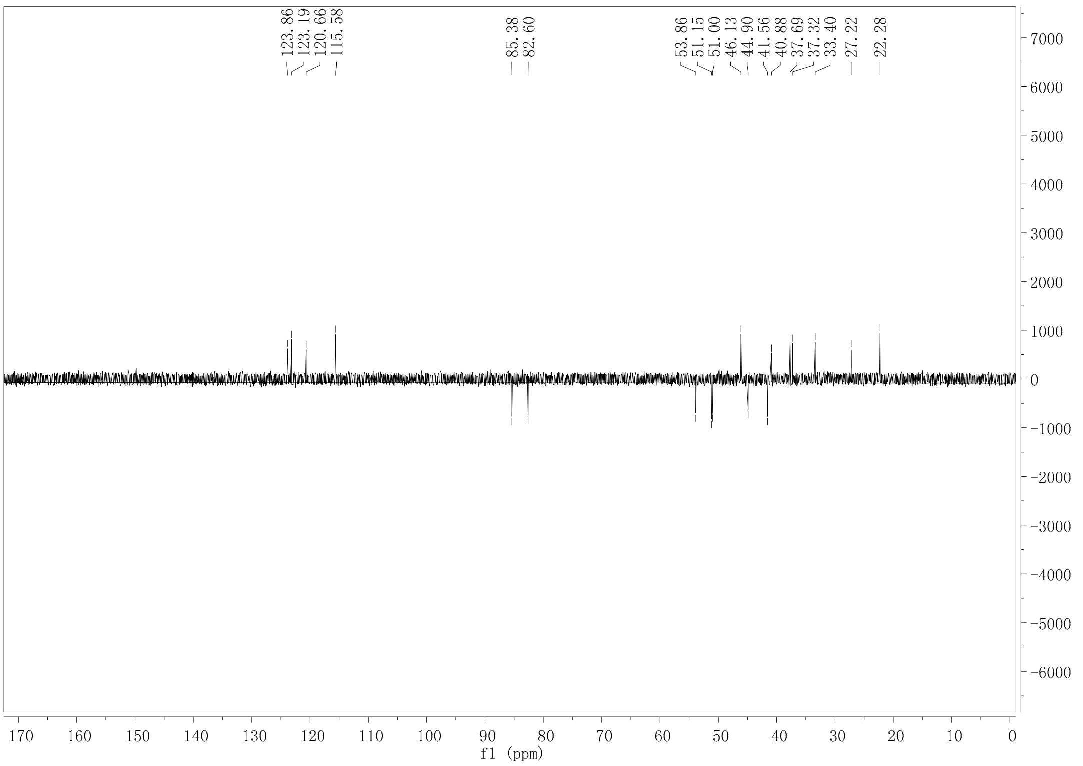

Figure S25. DEPT spectrum of compound **4** (150 MHz, in $CDCl_3$).

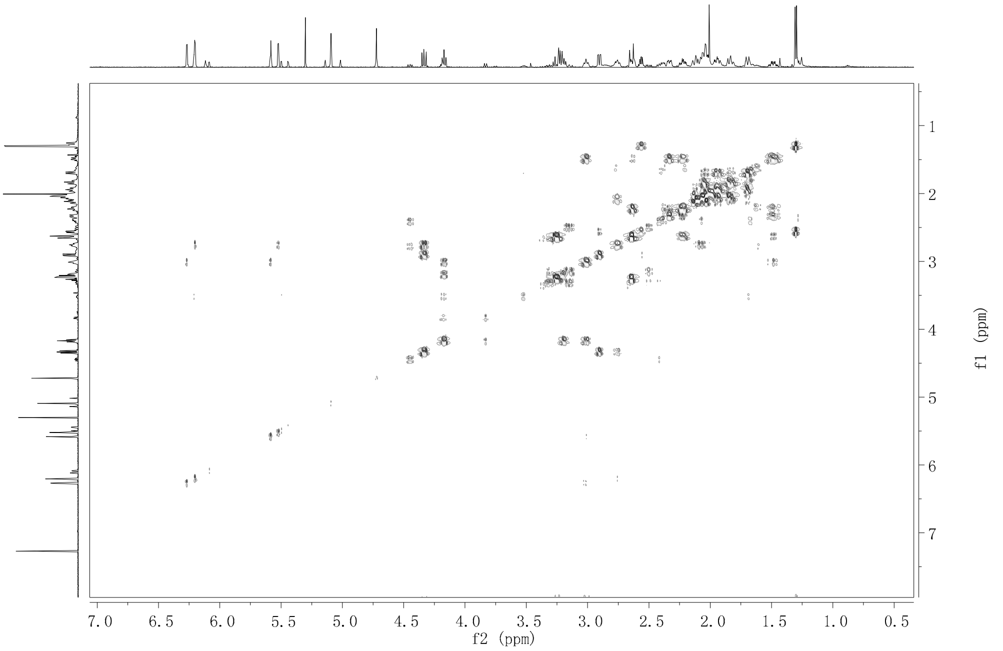

Figure S26. ^1H-^1H COSY spectrum of compound **4** (600 MHz, in $CDCl_3$).

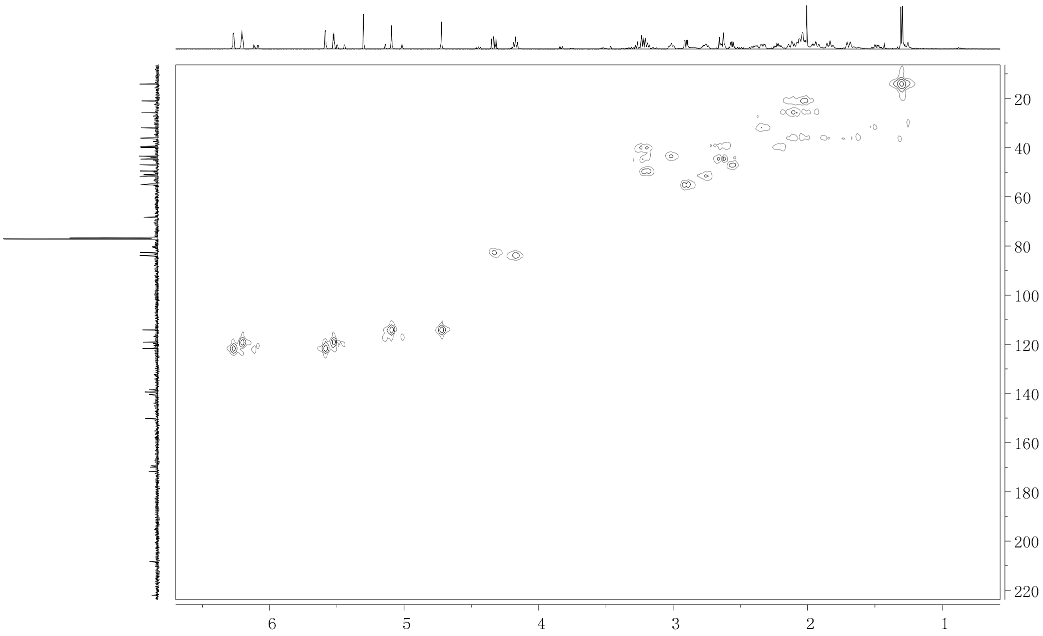

Figure S27. HSQC spectrum of compound **4** (600 MHz, in $CDCl_3$).

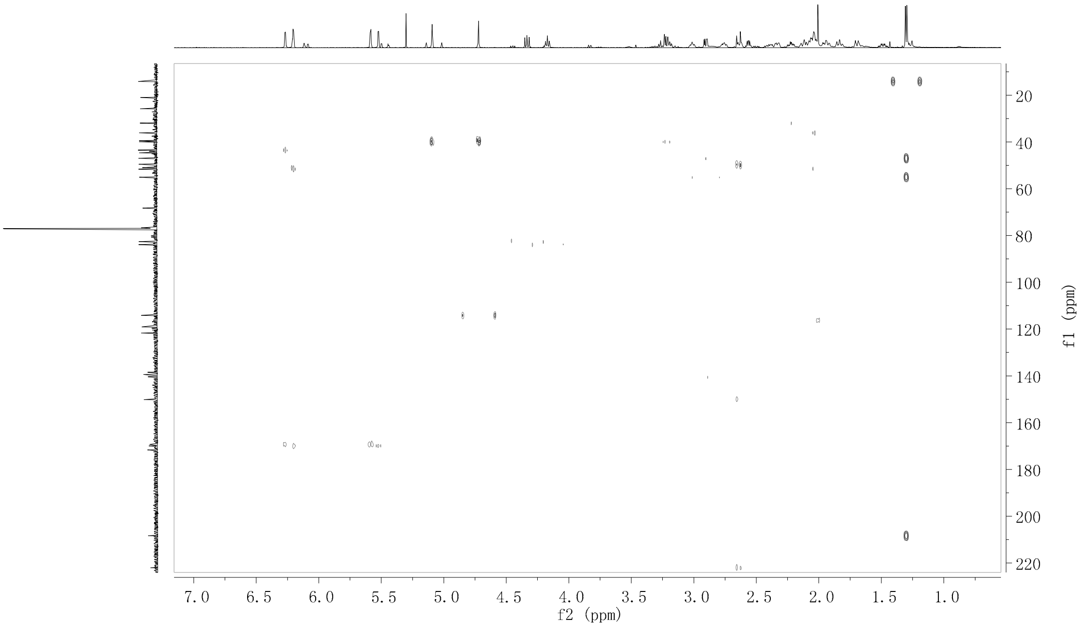

The acetone extract of *A. yunnanensis* and compounds **1**, **2**, **3**, **4** were analysised with the Diamonsil C18 column (250 mm × 4.6 mm, 5.0 μm, Dikma Technologies, Beijing, China), the mobile phase of HPLC-VWD as followed.

No.	Time (min)	Acetonitrile (%)	H_2O	Flow (mL/min)
1	0.00–30.00	40	60	1
2	0.00–35.00	40	60	1

Figure S28. HMBC spectrum of compound **4** (600 MHz, in $CDCl_3$).

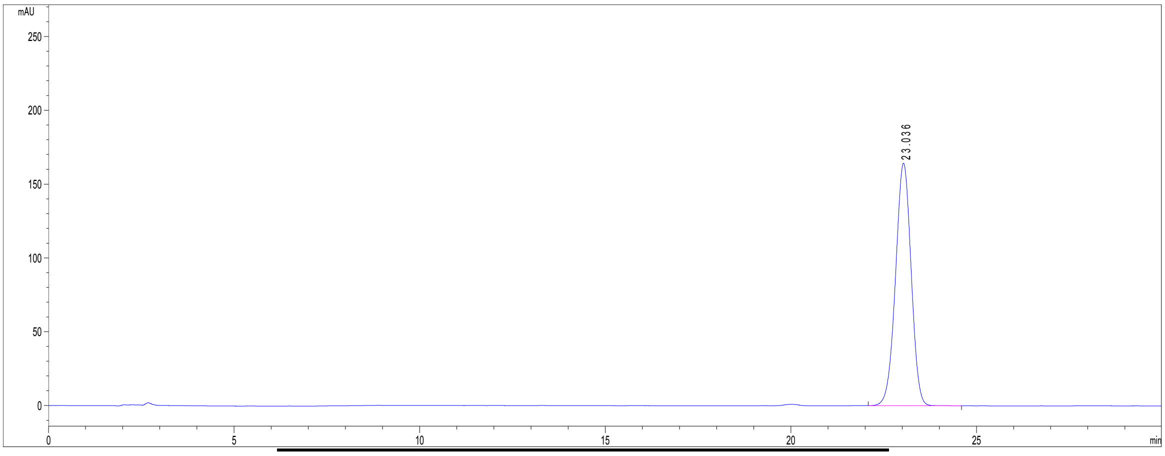

No.	Time	Area	Height	Width	Area%
1	23.036	4800.5	164.3	0.4545	100.000

Figure S29. HPLC profile of compound **1**. Mobile phase of 40% acetonitrile, Time = 30 min.

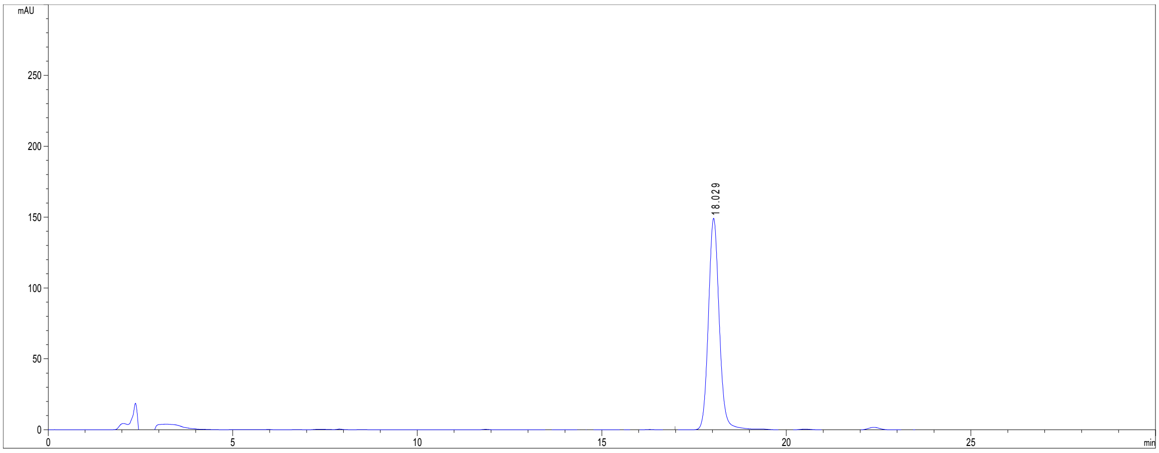

No.	Time
2-1	18.029

(**a**)

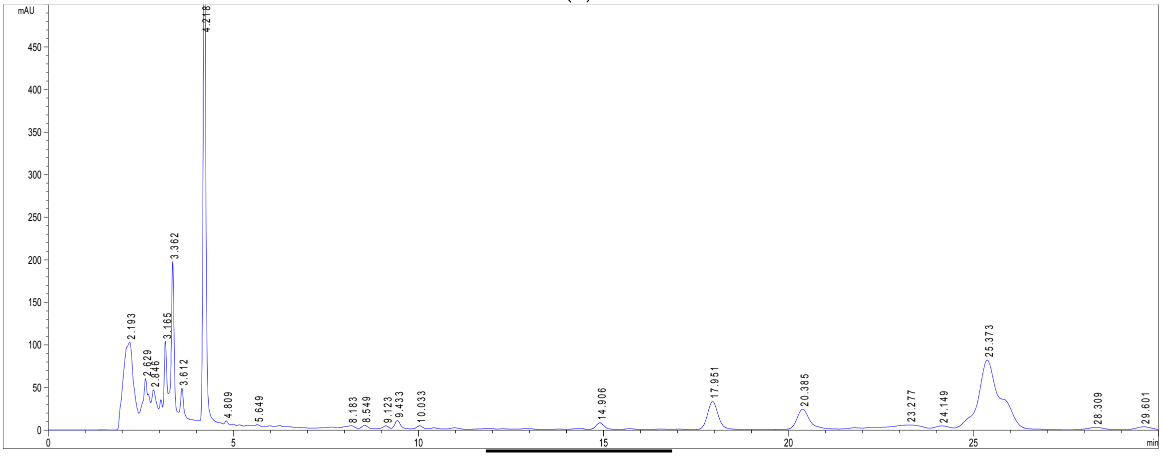

No.	Time
2-2	17.951

(**b**)

Figure S30. HPLC profile of compound **2** (**a**) and acetone extract of *A. yunnanensis* (**b**). Mobile phase of 40% acetonitrile, Time = 30 min. Compound **2** is a natural chemical constituent, which is identified by HPLC.

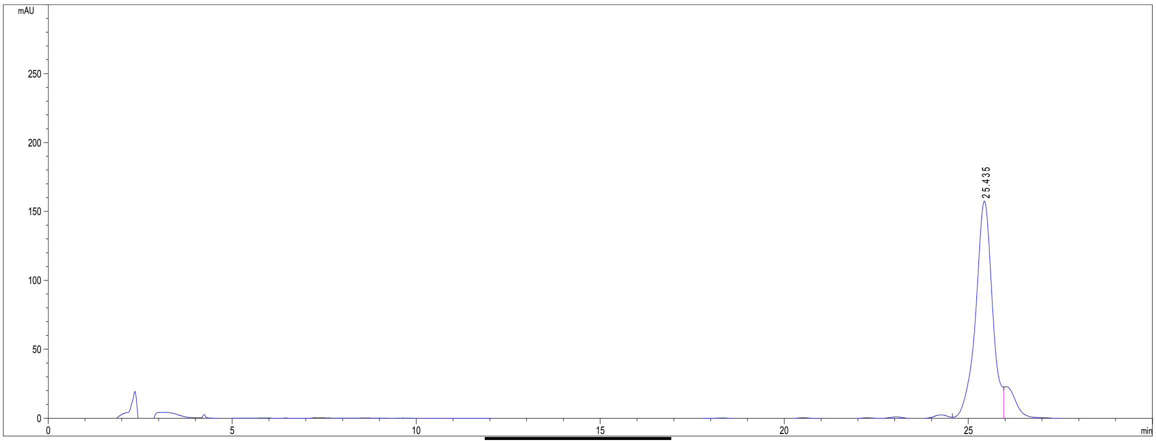

No.	Time
3-1	25.135

(**a**)

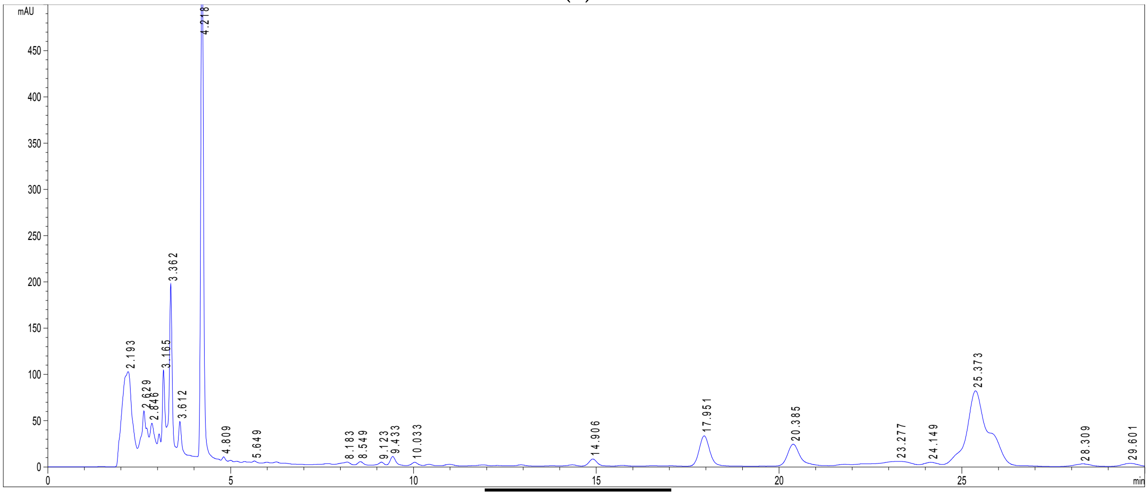

No.	Time
3-2	25.373

(**b**)

Figure S31. HPLC profile of compound **3** (**a**) and acetone extract of *A. yunnanensis* (**b**). Mobile phase of 40% acetonitrile, Time = 30 min. Compound **3** is a natural chemical constituent, which is identified by HPLC.

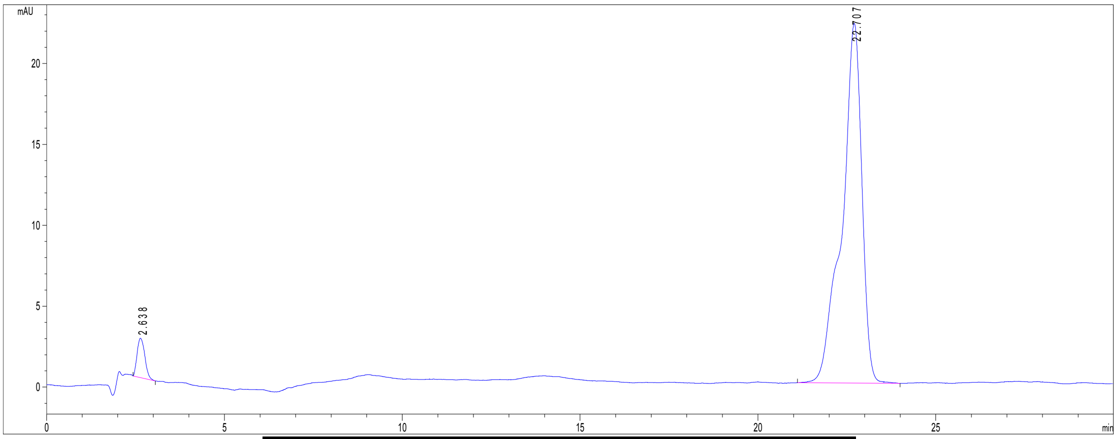

No.	Time	Area	Height	Width	Area%
4	22.707	863.2	22.2	0.5637	95.937

Figure S32. HPLC profile of compound **4**. Mobile phase of 40% acetonitrile, Time = 30 min.
